# Valence and core ionization energies of atoms and small molecules using variational Monte Carlo with explicit correlation and Slater-type basis sets

**DOI:** 10.1007/s00894-026-06987-y

**Published:** 2026-10-09

**Authors:** Josué de Jesus Oliveira Araújo, Pedro Oliveira Mariz de Carvalho, Rogério Custodio

**Affiliations:** Instituto de Química, Universidade Estadual de Campinas, P. O. Box 6154, Barão Geraldo, Campinas, São Paulo 13083-970 Brazil

**Keywords:** Variational Monte Carlo, Ionization energy, Slater-type basis functions, Boys–Handy explicit correlation function, Atomic and molecular calculations, Valence and core orbitals

## Abstract

**Context:**

Reliable prediction of both valence and core ionization energies remains challenging because these processes involve different balances of electron correlation, orbital relaxation, and basis-set flexibility. In this work, we assess whether variational Monte Carlo (VMC) with explicit correlation and compact Slater-type basis sets can provide accurate ionization energies without fitting to experimental data. As expected from their greater flexibility, double-zeta (DZ) and Hartree–Fock limit (HFL) Slater-type basis sets provide better agreement with experiment than single-zeta (SZ), particularly for atomic ionizations and some molecular cases. For H_2_O, VMC/HFL achieves a mean absolute error (MAE) of 0.42 eV, comparable to 0.39 eV from CCSD-GF/TZ2P; for CO, VMC/HFL gives an MAE of 0.67 eV. Removing the polarization functions has little effect on the core ionization energies. The calculations without polarization functions yield overall mean absolute errors of 0.65 eV for H_₂_O and 0.67 eV for CO. The accuracy arises from variational optimization of the explicit-correlation parameters without fitting to experimental ionization energies, supporting VMC as a practical approach for ionization-energy estimates and for extension to larger systems.

**Methods:**

Hartree–Fock wave functions with Slater-type basis sets were used as trial wave functions. For atoms, the SZ, DZ, and HFL orbital expansions were taken from the literature. For neutral molecules, Hartree–Fock calculations were performed with Gaussian 09 using Slater-type basis sets from the literature to generate the corresponding LCAO coefficients. Single valence and core ionization energies were computed with the VMC method using a nine-parameter Boys–Handy explicit correlation factor and Slater-type basis sets, with and without polarization functions. The VMC calculations and parameter optimizations were performed with computer programs developed by the authors’ research group.

**Graphical Abstract:**

**Figure:**
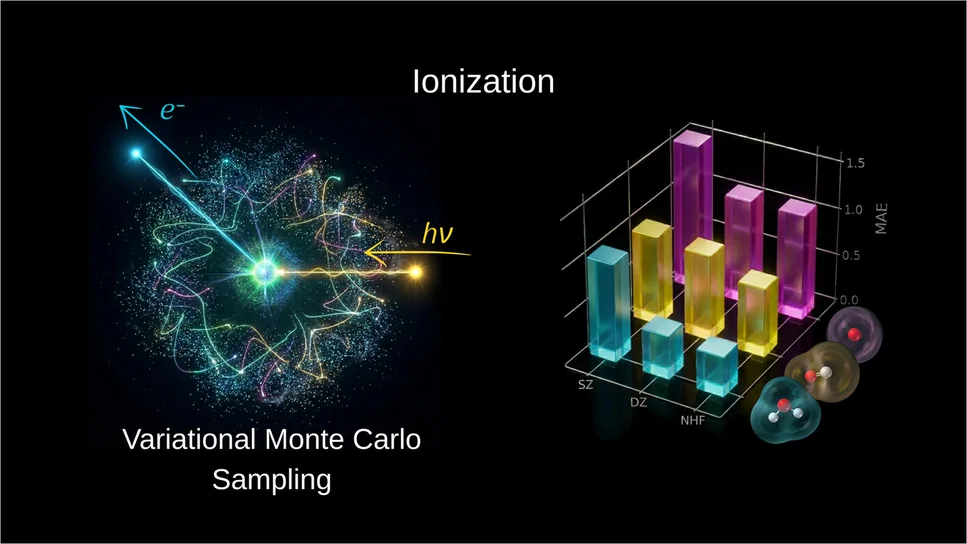

**Supplementary Information:**

The online version contains supplementary material available at https://doi.org/10.1007/s00894-026-06987-y.

## Introduction    

Accurate ionization energy calculations are challenging because the requirements depend on the region from which the electron is removed. Comparative studies, such as those by Lemierre et al. [1] and Ranasinghe et al. [2], show that this property can be computed at different levels of theory and computational cost. The simplest and most direct estimate comes from Koopmans’ theorem [3–5], which relates the ionization energy to the negative of the canonical orbital energy in the Hartree–Fock framework. Despite its simplicity and low computational cost, this approximation neglects electron correlation and orbital relaxation effects. Consequently, it provides reasonable values for valence ionizations, but its performance deteriorates markedly as the ionized orbital lies closer to the nucleus [3, 6].

Highly correlated ab initio methods, such as multireference configuration interaction (MRCI) [7–9], equation-of-motion coupled cluster (EOM-CC) [10, 11], and coupled cluster with single, double, and perturbative triple excitations (CCSD(T)) [2, 12], provide rigorous results but are computationally demanding and therefore restricted to small systems. The associated errors typically fall within ± 0.05 eV for valence ionizations and ± 0.5 eV for single core ionization [10].

Methods based on electron propagator theory (EPT) [13, 14] offer an efficient alternative for these calculations and can be viewed as a systematic way to incorporate correlation and orbital relaxation into Koopmans’ theorem through a diagrammatic expansion of the energy eigenvalue [15]. The outer valence Green’s function (OVGF) method [16], within the EPT framework, achieves good accuracy for valence electrons and enables substantial savings in computational time [17]. However, it yields reliable results mainly for ionizations involving valence electrons, which are less strongly bound to the system. Other EPT-based approaches, such as D2, P3, and P3 + [16], as well as newer-generation electron propagator methods [18], have been widely applied to molecular ionization energies. Reported errors range from − 0.3 to 1.7 eV, most frequently with absolute values below 0.5 eV [16]. Despite their usefulness, these methods are relatively complex in terms of theoretical development [19] and computational implementation.

For core-level ionizations, approaches such as the statistical averaged orbital potential method within density functional theory (DFT-SAOP) [20, 21] can provide accurate values and are available in some computational packages. However, the lack of uniformity among exchange–correlation functionals in DFT remains a limitation for its broader use.

Stochastic methods have also been applied to ionization energy studies, offering straightforward computational implementation and high accuracy. Among the quantum Monte Carlo formulations, diffusion Monte Carlo (DMC) can reach particularly high levels of accuracy [22]. In studies of single ionizations of small molecules, reported mean absolute deviations relative to experiment are below 5 eV for core ionizations and below 0.5 eV for the less energetic ionizations [21].

In contrast, variational quantum Monte Carlo (VMC), a simpler quantum Monte Carlo method, has received relatively little attention for this type of study. Its main advantages are the ease of implementation and the ability to treat explicit electron-correlation effects in a relatively straightforward way [22–25], even for systems containing many atoms. For example, Galek et al. [26] used Slater determinants and explicit electron-correlation functions in DMC and VMC calculations for a set of small molecules and recovered more than 40% of the correlation energy with VMC and more than 80% with DMC. Their valence single-ionization energies showed absolute errors below 0.2 eV. Drummond et al. [27] studied Ne and Ne⁺ with these methods and obtained, in the worst VMC cases, an error of 0.434% when all electrons were included in the description of the systems, and 0.603% when a pseudopotential was employed to compute the ionization energy. Maldonato et al. [28] investigated single ionization energies from lithium to argon and found absolute errors below 0.6 eV when VMC was used with single-configuration wave functions, and below 0.3 eV when multiconfigurational wave functions were adopted. Seth et al. [29] examined first-row atoms and reported a mean absolute deviation of 0.023(2) eV and a maximum absolute deviation of 0.076(5) eV. Other recent studies have also successfully applied VMC to treat explicit correlation and optimize complex wave functions [30–32].

In terms of computational cost, conventional coupled-cluster methods exhibit steep formal scaling with the size of the orbital space, reaching $\mathcal{O}\left({M}^{6}\right)$ for CCSD and $\mathcal{O}\left({M}^{8}\right)$ for CCSDT, where M denotes a measure of the orbital-space or basis-set size. More specifically, the actual computational cost depends strongly on the numbers of occupied and virtual orbitals [16]. Similarly, conventional second- and third-order algebraic-diagrammatic construction methods typically scale as $\mathcal{O}\left({M}^{5}\right)$ and $\mathcal{O}\left({M}^{6}\right)$, respectively [33, 34]. By contrast, conventional VMC implementations typically exhibit a formal scaling of approximately $\mathcal{O}\left({N}_{e}^{3}\right)$ to $\mathcal{O}\left({N}_{e}^{4}\right)$ with the number of electrons (${N}_{e}$), although the actual scaling depends on the form of the trial wave function, the optimization procedure, and the specific implementation [35]. Because these scaling estimates are expressed in terms of different variables, their exponents alone do not provide a direct quantitative comparison of computational cost. Nevertheless, although VMC is not independent of basis-set size, it avoids the explicit treatment of large virtual-orbital spaces and may therefore offer practical computational advantages for calculations involving larger basis sets and systems.

Given the reduced use of variational Monte Carlo for ionization-energy calculations and its straightforward computational implementation, the goal of the present work is to systematically assess the performance of the VMC method for computing single valence and core ionization energies of atoms and some molecules. We examine the effects of the basis set and of a nine-parameter Boys–Handy explicit correlation function on the calculated ionization energies.

## Computational methods

Variational Monte Carlo [29, 36] is based on the application of the expectation value theorem and the variational principle to compute the energy of a given system and to minimize this quantity in order to optimize intrinsic parameters of the wave function. The mean value of the energy, $\left\langle E\right\rangle=\left\langle\Psi\left|\widehat H\right|\Psi\right\rangle/\left\langle\Psi\left|\Psi\right.\right\rangle$ , is evaluated by computing the local energy, defined as, $E_L=\widehat H\Psi/\Psi$, and sampling the 3 N coordinates of the N electrons using importance sampling generated with the Metropolis algorithm [37, 38]. A major advantage of VMC is that the local energy can be written as:

1 $$E_L=\frac{\widehat{T\;}\Psi}\Psi+V$$

Unlike conventional electronic-structure methods, the most demanding term is the application of the kinetic energy operator ($\hat{T}$) to the trial wave function, whereas the potential energy (V) is given directly by its classical expression. This feature makes the VMC algorithm relatively simple to implement computationally. However, VMC depends strongly on the quality of the trial wave function, which is usually constructed prior to the calculation.

To ensure that the electronic properties of the systems studied are properly described within VMC, the wave function must be antisymmetric with respect to the exchange of electrons among spin orbitals. A few studies have explored the use of density-matrix theory to obtain an exact representation of exchange effects [39–42]. However, the simplest approach assumes a wave function given by the product of one determinant containing only-spin electrons and another containing only β-spin electrons, i.e.,$\Psi=\Psi_a\Psi_\beta$. In the present work, this form was adopted using orbital functions obtained from Hartree–Fock calculations with Slater-type basis sets taken from the literature [43–46]. Single-zeta (SZ), double-zeta (DZ), and Hartree–Fock limit (HFL) basis sets were employed.

Electronic-correlation effects were included through the explicit Boys–Handy correlation function [24, 26], which multiplies the Hartree–Fock wave function:

2 $$\Psi_{VMC}=\Psi_a\Psi_\beta\Psi_{corr.}$$

The explicit correlation factor used here has the form:

3 $${\Psi}_{corr.}=\mathrm{exp}\left({\sum}_{A, i>j}^{n}{U}_{A, ij}\right)$$

where:

$$U_{Aij=}c_{1A}{\bar{r}}_{ij}+c_{2A}{\bar{r}_{ij}^{2}}+c_{3A}{\bar{r}_{ij+}^{3}}c_{4A}{\bar{r}_{ij}^{4}}+c_{5A}\left({\bar{r}_{iA}^{2}}+{\bar{r}_{jA}^{2}}\right)+c_{6A}\left({\bar{r}_{iA}^{3}}+{\bar{r}_{jA}^{3}}\right)+$$

4 $$+c_{7A}\left(\bar{r}_{iA}^{4}+\bar{r}_{jA}^{4}\right)+C_{8A}\left({\bar{r}_{iA}^{2}}{\bar{r}_{jA}^{2}}\right)+C_{9A}\left({\bar{r}_{iA}^{2}}+{\bar{r}_{jA}^{2}}\right)\bar{r}_{ij}$$

with:

5 $${\bar{r}}_{iA}=\frac{b_Ar_{iA}}{1+b_Ar_{iA}},{\bar{r}}_{ij}=\frac{d_Ar_{ij}}{1+d_Ar_{il}}$$

The first four terms in Eq. (4) represent electron–electron dynamic-correlation corrections; the fifth, sixth, and seventh terms account for electron–nucleus correlation; and the eighth and ninth terms describe electron–electron–nucleus correlation effects [26]. The coefficients were optimized variationally.

For the atomic systems and their ions, the correlation function parameters were optimized, and the value adopted for each parameter was taken as the average over the last 20 cycles of the optimization procedure using the Newton–Raphson method [47]. In total, 45 cycles were performed: the first 20 used 1000 Monte Carlo steps, and the final 25 used 20,000 steps. After optimization, a final stage was carried out with 200,000-step calculations. All calculations for these systems employed 100 walkers. For the molecules and their ions, a preliminary optimization of 80 cycles was performed using the Nelder–Mead simplex method [48] to approach the global minimum, followed by a shorter refinement using the Newton–Raphson method [47] to further improve the minimum. Once the optimized parameters were obtained, a final VMC simulation with 700,000 steps was performed.

The wave functions for the neutral molecules and atoms were used as starting points to obtain the corresponding wave functions for the ionized systems. For molecules, these wave functions were extracted from output files generated by the electronic structure calculation software Gaussian 09 [49]. Ionization energies were computed as differences between the electronic energies of the ionized and neutral species [13]. These energies were computed using the VMC method with code developed by the authors’ research group. For theoretical cationic states that comprise more than one component, the corresponding energies were obtained as the average of the cationic states generated by removing an electron from the neutral ground state. Similarly, when experimental spectroscopy data contained more than one cationic state, the experimental ionization energies were determined from the weighted mean of those states, using the coefficients of fractional parentage [50, 51] with respect to the neutral ground state. When optical spectroscopy data were unavailable, they were combined with x-ray spectroscopy data to obtain the ionization energies.

## Results and discussion

### Atomic ionization energies

Tables 1 and 2 show the calculated and experimental ionization energies [51–54] for atoms from He to Ne and from Na to Ar, respectively, along with the relative errors with respect to experiment. Subscripts on the element symbols indicate the ionized orbitals. When a spectroscopic term is not specified, the reported value corresponds to an ionization energy obtained by combining ionic microstates, as described in the previous section.

**Table 1:** Calculated and experimental ionization energies (eV) for atoms from He to Ne, with relative deviations. Values in parentheses are the relative deviations; subscripts on the element symbols denote the ionized orbitals

Ion	SZ	DZ	HFL	Experimental
${\mathrm{H}\mathrm{e}}_{1s}^{+}{}^{2}S$	24.555 (−0.13%)	24.577 (−0.04%)	24.579 (−0.03%)	24.59^a^
${\mathrm{L}\mathrm{i}}_{1s}^{+}{}^{3}S$	64.437 (0.04%)	64.395 (−0.03%)	64.412 (0.001%)	64.41^a^
${\mathrm{L}\mathrm{i}}_{1s}^{+}$	64.942 (0.08%)	64.822 (−0.10%)	64.823 (−0.10%)	64.89^a^
${\mathrm{L}\mathrm{i}}_{2s}^{+}{}^{1}S$	5.328 (−1.19%)	5.320 (−1.32%)	5.314 (−1.44%)	5.39^a^
${\mathrm{B}\mathrm{e}}_{1s}^{+}{}^{2}S$	124.177 (0.44%)	124.130 (0.40%)	124.122 (0.40%)	123.6^a^
${\mathrm{B}\mathrm{e}}_{2s}^{+}{}^{2}S$	8.734 (−6.31%)	8.728 (−6.38%)	8.733 (−6.33%)	9.32^a^
${\mathrm{B}}_{1s}^{+}{}^{3}P$	201.89 (0.60%)	201.418 (0.36%)	201.402 (0.36%)	200.7^a^
${\mathrm{B}}_{1s}^{+}$	202.17 (0.49%)	201.858 (0.34%)	201.864 (0.34%)	201.18^a^
${\mathrm{B}}_{2s}^{+}{}^{3}P$	12.20 (−5.61%)	12.360 (−4.39%)	12.332 (−4.61%)	12.93^a^
${\mathrm{B}}_{2s}^{+}$	13.36 (−4.89%)	13.482 (−4.01%)	13.459 (−4.17%)	14.04^a^
${\mathrm{B}}_{2p}^{+}{}^{1}S$	7.930 (−4.41%)	8.498 (2.42%)	8.455 (1.91%)	8.30^a^
${\mathrm{C}}_{1s}^{+}$	297.97 (0.40%)	297.719 (0.31%)	297.718 (0.31%)	296.8^b^
${\mathrm{C}}_{2s}^{+}{}^{4}P$	15.83 (−4.59%)	15.915 (−4.08%)	15.930 (−3.99%)	16.59^a^
${\mathrm{C}}_{2s}^{+}$	18.53 (−4.41%)	18.726 (−3.41%)	18.748 (−3.30%)	19.39^a^
${\mathrm{C}}_{2p}^{+}{}^{2}P$	10.70 (−4.97%)	11.436 (1.54%)	11.443 (1.61%)	11.26^a^
${\mathrm{N}}_{1s}^{+}$	412.03 (0.52%)	411.969 (0.50%)	411.890 (0.49%)	409.9^b^
${\mathrm{N}}_{2s}^{+}{}^{5}S$	19.36 (−4.78%)	19.641 (−3.41%)	19.655 (−3.34%)	20.33^a^
${\mathrm{N}}_{2s}^{+}$	24.49 (−3.47%)	24.722 (−2.56%)	24.747 (−2.47%)	25.37^a^
${\mathrm{N}}_{2p}^{+}{}^{3}P$	14.08 (−3.18%)	14.725 (1.24%)	14.719 (1.20%)	14.55^a^
${\mathrm{O}}_{1s}^{+}$	546.32 (0.59%)	546.01 (0.54%)	546.140 (0.56%)	543.1^b^
${\mathrm{O}}_{2s}^{+}{}^{4}P$	28.16 (−1.12%)	28.37 (−0.36%)	28.427 (−0.18%)	28.48^a^
${\mathrm{O}}_{2s}^{+}$	31.95 (−1.12%)	32.26 (−0.15%)	32.351 (0.13%)	32.31^a^
${\mathrm{O}}_{2p}^{+}{}^{4}S$	13.06 (−4.04%)	13.48 (−0.98%)	13.635 (0.20%)	13.61^a^
${\mathrm{O}}_{2p}^{+}$	15.56 (−4.24%)	16.18 (−0.43%)	16.301 (0.33%)	16.25^a^
${\mathrm{F}}_{1s}^{+}$	699.06 (0.34%)	698.62 (0.27%)	698.79 (0.30%)	696.71^b^
${\mathrm{F}}_{2s}^{+}{}^{3}P$	37.75 (−0.29%)	38.41 (1.46%)	38.60 (1.97%)	37.86^a^
${\mathrm{F}}_{2s}^{+}$	39.96 (−0.54%)	40.63 (1.13%)	40.84 (1.65%)	40.17^a^
${\mathrm{F}}_{2p}^{+}{}^{3}P$	16.56 (−4.96%)	17.38 (−0.29%)	17.59 (0.91%)	17.43^a^
${\mathrm{F}}_{2p}^{+}$	17.92 (−3.92%)	18.58 (−0.37%)	18.81 (0.86%)	18.65^a^
${\mathrm{N}\mathrm{e}}_{1s}^{+}{}^{2}S$	870.04 (−0.04%)	869.60 (−0.09%)	869.67 (−0.08%)	870.37^b^
${\mathrm{N}\mathrm{e}}_{2s}^{+}{}^{2}S$	48.50 (0.06%)	49.70 (2.52%)	49.84 (2.82%)	48.48^a^
${\mathrm{N}\mathrm{e}}_{2p}^{+}{}^{2}P$	20.86 (−3.42%)	21.47 (−0.57%)	21.68 (0.40%)	21.60^a^

^a^From optical data [54]

^b^From optical and x-ray data combined [51–54]

**Table 2:** Calculated and experimental ionization energies (eV) for atoms from Na to Ar, with relative deviations. Values in parentheses are the relative deviations; subscripts on the element symbols denote the ionized orbitals

Ion	SZ	DZ	HFL	Experimental
${\mathrm{N}\mathrm{a}}_{1s}^{+}$	1079.96 (0.09%)	1078.09 (−0.08%)	1078.35 (−0.06%)	1079^b^
${\mathrm{N}\mathrm{a}}_{2s}^{+}{}^{3}S$	72.52 (2.20%)	71.79 (1.16%)	72.08 (1.57%)	70.96^a^
${\mathrm{N}\mathrm{a}}_{2s}^{+}$	72.91 (2.59%)	72.00 (1.31%)	72.28 (1.71%)	71.07^a^
${\mathrm{N}\mathrm{a}}_{2p}^{+}{}^{3}P$	39.02 (2.58%)	38.18 (0.38%)	38.41 (0.99%)	38.0^a^
${\mathrm{N}\mathrm{a}}_{2p}^{+}$	39.06 (2.42%)	38.27 (0.32%)	38.50 (0.94%)	38.1^a^
${\mathrm{N}\mathrm{a}}_{3s}^{+}{}^{1}S$	4.98 (−3.19%)	4.86 (−5.42%)	5.08 (−1.25%)	5.14^a^
${\mathrm{M}\mathrm{g}}_{1s}^{+}{}^{2}S$	1310.18 (−0.05%)	1309.97 (−0.07%)	1309.89 (−0.08%)	1311^b^
${\mathrm{M}\mathrm{g}}_{2s}^{+}{}^{2}S$	96.70 (−0.14%)	97.42 (0.60%)	97.49 (0.67%)	96.8^b^
${\mathrm{M}\mathrm{g}}_{2p}^{+}{}^{2}P$	56.98 (−1.15%)	58.03 (0.67%)	58.05 (0.71%)	57.64^a^
${\mathrm{M}\mathrm{g}}_{3s}^{+}{}^{2}S$	6.21 (−18.75%)	7.32 (−4.26%)	7.12 (−6.87%)	7.65^a^
${\mathrm{A}\mathrm{l}}_{1s}^{+}$	1564.81 (−0.14%)	1566.45 (−0.04%)	1566.45 (−0.04%)	1567.0^b^
${\mathrm{A}\mathrm{l}}_{2s}^{+}$	124.59 (−0.81%)	127.36 (1.40%)	127.38 (1.42%)	125.6^b^
${\mathrm{A}\mathrm{l}}_{2p}^{+}$	80.03 (−0.59%)	82.36 (2.32%)	82.26 (2.19%)	80^b^
${\mathrm{A}\mathrm{l}}_{3s}^{+}{}^{3}P$	8.68 (−18.32%)	10.09 (−5.05%)	10.01 (−5.85%)	10.63^a^
${\mathrm{A}\mathrm{l}}_{3s}^{+}$	9.63 (−14.90%)	10.84 (−4.28%)	10.80 (−4.57%)	11.32^a^
${\mathrm{A}\mathrm{l}}_{3p}^{+}{}^{1}S$	4.61 (−22.85%)	5.87 (−1.73%)	5.81 (−2.80%)	5.98^a^
${\mathrm{S}\mathrm{i}}_{1s}^{+}$	1844.3 (−0.09%)	1845.71 (−0.02%)	1845.83 (−0.01%)	1846^b^
${\mathrm{S}\mathrm{i}}_{2s}^{+}$	157.41 (0.90%)	160.28 (2.74%)	160.34 (2.78%)	156^b^
${\mathrm{S}\mathrm{i}}_{2p}^{+}$	107.3 (0.80%)	109.41 (2.74%)	109.50 (2.81%)	106^b^
${\mathrm{S}\mathrm{i}}_{3s}^{+}{}^{4}P$	10.9 (−18.89%)	12.73 (−5.44%)	12.89 (−4.27%)	13.46^a^
${\mathrm{S}\mathrm{i}}_{3s}^{+}$	12.8 (−15.33%)	14.58 (−3.79%)	14.71 (−2.93%)	15.16^a^
${\mathrm{S}\mathrm{i}}_{3p}^{+}{}^{2}P$	8.2 (0.42%)	8.07 (−1.09%)	8.26 (1.21%)	8.16^a^
${\mathrm{P}}_{1s}^{+}$	2155.9 (0.09%)	2148.09 (−0.27%)	2148.08 (−0.28%)	2154^b^
${\mathrm{P}}_{2s}^{+}$	190.6 (−1.76%)	196.20 (1.13%)	196.05 (1.06%)	194^b^
${\mathrm{P}}_{2p}^{+}$	138.3 (−1.59%)	139.52 (−0.70%)	139.52 (−0.70%)	140^b^
${\mathrm{P}}_{3s}^{+}{}^{5}S$	14.2 (−11.88%)	15.26 (−5.56%)	15.50 (−4.06%)	16.15^a^
${\mathrm{P}}_{3s}^{+}$	17.7 (−7.53%)	18.55 (−3.14%)	18.72 (−2.28%)	19.15^a^
${\mathrm{P}}_{3p}^{+}{}^{3}P$	10.6 (0.86%)	10.48 (−0.46%)	10.67 (1.39%)	10.53^a^
${\mathrm{S}}_{1s}^{+}$	2472.8 (−0.17%)	2473.90 (−0.13%)	2474.04 (−0.12%)	2477^b^
${\mathrm{S}}_{2s}^{+}$	230.7 (−1.83%)	235.53 (0.22%)	235.61 (0.26%)	235^b^
${\mathrm{S}}_{2p}^{+}$	170.3 (−0.09%)	172.99 (1.46%)	173.22 (1.59%)	170^b^
${\mathrm{S}}_{3s}^{+}{}^{4}P$	20.1 (−0.53%)	20.68 (2.33%)	20.78 (2.86%)	20.21^a^
${\mathrm{S}}_{3s}^{+}$	22.5 (−2.66%)	23.00 (−0.28%)	23.25 (0.77%)	23.07^a^
${\mathrm{S}}_{3p}^{+}{}^{4}S$	9.6 (−7.02%)	10.07 (−2.53%)	10.00 (−3.25%)	10.34^a^
${\mathrm{S}}_{3p}^{+}$	10.8 (−8.85%)	11.70 (−1.37%)	11.68 (−1.55%)	11.87^a^
${\mathrm{C}\mathrm{l}}_{1s}^{+}$	2821.7 (−0.29%)	2823.65 (−0.22%)	2823.67 (−0.22%)	2830^b^
${\mathrm{C}\mathrm{l}}_{2s}^{+}$	273.2 (−1.71%)	278.59 (0.21%)	278.62 (0.22%)	278^b^
${\mathrm{C}\mathrm{l}}_{2p}^{+}$	207.1 (−0.92%)	210.29 (0.62%)	210.35 (0.65%)	209^b^
${\mathrm{C}\mathrm{l}}_{3s}^{+}{}^{3}P$	26.2 (6.92%)	27.12 (10.49%)	26.94 (9.75%)	24.55^a^
${\mathrm{C}\mathrm{l}}_{3s}^{+}$	27.6 (9.36%)	28.41 (12.63%)	28.25 (11.96%)	25.23^a^
${\mathrm{C}\mathrm{l}}_{3p}^{+}{}^{3}P$	12.7 (−2.08%)	13.04 (0.49%)	12.93 (−0.37%)	12.97^a^
${\mathrm{C}\mathrm{l}}_{3p}^{+}$	13.0 (−5.16%)	13.76 (0.68%)	13.74 (0.54%)	13.67^a^
${\mathrm{A}\mathrm{r}}_{1s}^{+}{}^{2}S$	3192.4 (−0.42%)	3195.78 (−0.32%)	3195.47 (−0.33%)	3205.9^b^
${\mathrm{A}\mathrm{r}}_{2s}^{+}{}^{2}S$	317.0 (−2.85%)	324.45 (−0.57%)	324.15 (−0.66%)	326.3^b^
${\mathrm{A}\mathrm{r}}_{2p}^{+}{}^{2}P$	245.9 (−1.33%)	250.50 (0.54%)	249.98 (0.33%)	249.15^b^
${\mathrm{A}\mathrm{r}}_{3s}^{+}{}^{2}S$	31.8 (8.66%)	33.62 (14.99%)	33.03 (12.97%)	29.24^a^
${\mathrm{A}\mathrm{r}}_{3p}^{+}{}^{2}P$	14.2 (−10.14%)	16.35 (3.75%)	15.77 (0.05%)	15.76^a^

^a^From optical data [54];

^b^From optical and x-ray data combined [51–54]

As can be seen from Tables 1 and 2, the absolute relative errors obtained with the SZ basis set are systematically larger than those obtained with the DZ and HFL basis sets. The latter two basis sets yield very similar results, with most corresponding DZ and HFL ionization energies differing from each other by no more than 0.20 eV. Restricting the comparison to DZ and HFL, the largest deviations occur for ${\mathrm{C}\mathrm{l}}_{3s}^{+}({}^{3}P)$, ${\mathrm{C}\mathrm{l}}_{3s}^{+}$, and ${\mathrm{A}\mathrm{r}}_{3s}^{+}({}^{2}S)$, with absolute relative errors between 10 and 15%. Several other 3s ions (${\mathrm{P}}_{3s}^{+}({}^{5}P)$, ${\mathrm{S}\mathrm{i}}_{3s}^{+}({}^{4}P)$, ${\mathrm{A}\mathrm{l}}_{3s}^{+}({}^{3}P)$ and ${\mathrm{M}\mathrm{g}}_{3s}^{+}({}^{2}S)$) show errors close to 5%. The 2s ions of the second period exhibit similar behavior, with errors of about 6% for ${\mathrm{B}\mathrm{e}}_{2s}^{+}({}^{2}S)$ and about 4% for ${\mathrm{B}}_{2s}^{+}({}^{3}P)$, ${\mathrm{B}}_{2s}^{+}$, ${\mathrm{C}}_{2s}^{+}({}^{4}P)$, ${\mathrm{C}}_{2s}^{+}$, ${\mathrm{N}}_{2s}^{+}({}^{5}P)$ and ${\mathrm{N}}_{2s}^{+}$. For the remaining ions, the errors are smaller, with most values below 1%.

Particularly noteworthy is the close agreement between the values obtained with the DZ and HFL basis sets for the atoms from He to Ne listed in Table 1, especially for ${\mathrm{H}\mathrm{e}}_{1s}^{+}({}^{2}S)$, ${\mathrm{L}\mathrm{i}}_{1s}^{+}({}^{3}S)$, ${\mathrm{B}\mathrm{e}}_{1s}^{+}({}^{2}S)$, and ${\mathrm{C}}_{1s}^{+}$. The largest DZ–HFL difference is 0.23 eV for${\mathrm{F}}_{2p}^{+}$, followed by differences of 0.21 eV for ${\mathrm{F}}_{2s}^{+}({}^{3}P)$, ${\mathrm{F}}_{2p}^{+}({}^{3}P)$, and ${\mathrm{N}\mathrm{e}}_{2p}^{+}\left({}^{2}P\right)$. Overall, the DZ and HFL values differ by no more than 0.20 eV for 28 of the 32 ionizations in Table 1.

Table 3 summarizes the mean absolute errors for the atoms, grouped by period of the periodic table and by the orbitals from which electrons are removed, for each basis-set level. The contrast between SZ and DZ/HFL is clear: the SZ averages are systematically larger, whereas the DZ and HFL mean absolute errors are very close to each other regardless of the grouping. The averages for third-period atoms are about three times larger than those for atoms in periods 1 and 2, largely due to inner-shell 1s ionizations, with errors reaching ~ 10 eV for ${\mathrm{A}\mathrm{r}}_{1s}^{+}({}^{2}S$). This behavior is also evident in the orbital-based grouping in Table 3, which shows mean absolute errors near 2 eV for the 1s orbital and about 1 eV for the 2s and 3s orbitals. The smallest mean absolute errors are obtained for the 3p orbitals. This may reflect the more balanced description of the neutral and cationic states in these outer-valence ionizations, for which removal of an electron generally induces less orbital relaxation and a smaller change in electron correlation than inner-shell ionization. Consequently, errors in the total energies of the neutral and cationic states may cancel more effectively when the ionization energy is evaluated as an energy difference.

**Table 3:** Mean absolute errors (eV) of the ionization energies relative to experiment, grouped by period and by the ionized orbital, for the SZ, DZ, and HFL basis sets

Group	SZ	DZ	HFL
Periods 1 and 2	0.74	0.58	0.59
Period 3	2.11	1.48	1.47
Orbital 1s	2.39	2.04	2.04
Orbital 2s	1.56	0.93	0.99
Orbital 2p	0.97	0.74	0.75
Orbital 3s	1.60	1.16	1.06
Orbital 3p	0.73	0.18	0.13
Total	1.56	1.12	1.12

### Molecular ionization energies

Table 4 lists the ionization energies of the water molecule obtained with the VMC method using SZ, DZ, and HFL basis sets (all with polarization functions), together with CCSD-GF/TZ2P literature values [55] and the corresponding experimental data [56]. The results indicate that VMC with the HFL basis set yields the best value for the ${1a}_{1}$ orbital, which is closer to experimental value than the CCSD-GF result. The SZ and DZ basis sets lead to larger, yet still acceptable, errors, particularly given that core ionization involves substantial relaxation effects [55]. The absolute (relative) errors are 1.41 eV (0.26%), 0.15 eV (0.03%), and 0.09 eV (0.02%) for SZ, DZ, and HFL, respectively. Both electron correlation and relativistic effects can influence ionization energies through their different contributions to the neutral and cationic states. The present VMC calculations recover a significant fraction of the correlation energy but do not include relativistic corrections or explicit orbital reoptimization. State-specific optimization of the explicit-correlation function provides additional flexibility to describe differences between the neutral and cationic states, but its effect cannot be separated from possible error cancellation in the calculated ionization energies. Therefore, the close agreement obtained for some inner-shell ionizations should be interpreted cautiously. A quantitative decomposition of correlation, orbital-relaxation, and relativistic effects would require additional calculations beyond the scope of the present study.

**Table 4:** Single ionization energies (eV) of H_2_O obtained with VMC using the SZ, DZ, and HFL basis set (all with polarization functions) and CCSD-GF calculations. Values in parentheses are the absolute errors with respect to experiment. MAE denotes the mean absolute error

Ionization	VMC-SZ	VMC-DZ	VMC-HFL	CCSD-GF [55]	Experiment [56]
1a_1_	541.11 (1.41)	539.85 (0.15)	539.61 (0.09)	541.02 (1.32)	539.70
2a_1_	34.63 (2.02)	34.45 (1.85)	34.30 (1.69)	32.49 (0.12)	32.61
1b_2_	18.06 (0.68)	18.62 (0.11)	18.77 (0.26)	18.86 (0.12)	18.51
3a_1_	15.26 (0.51)	14.88 (0.14)	14.78 (0.04)	14.61 (0.14)	14.74
1b_1_	13.19 (0.58)	12.59 (0.03)	12.61 (0.01)	12.32 (0.29)	12.62
MAE	1.04	0.46	0.42	0.39	

For the remaining ionizations, except those originating from the ${1\mathrm{b}}_{1}$ and ${3\mathrm{a}}_{1}$ orbitals, the deviations obtained in the VMC simulations are larger than those from CCSD-GF. Even so, the absolute errors remain within acceptable limits. The most discrepant case is the ionization of the ${2\mathrm{a}}_{1}$ orbital, with deviations of 2.02 eV (SZ), 1.85 eV (DZ), and 1.69 eV (HFL), in contrast to the 0.12 eV error obtained with CCSD-GF. This difference can be attributed to the character of the ${2\mathrm{a}}_{1}$ orbital, which lies between the valence and inner-shell regions, an intermediate zone marked by strong electron correlation and significant coupling to multiconfigurational electronic states. Because the explicit correlation function used in VMC primarily recovers dynamic correlation, the presence of static correlation is expected to contribute to the larger error observed.

Overall, the VMC calculations with the DZ and HFL basis sets show good agreement with experiment, with mean absolute errors (MAEs) of 0.46 and 0.42 eV, respectively, close to the CCSD-GF deviation of 0.39 eV. Although CCSD-GF provides slightly better agreement with experiment, its formal scaling with the size of the orbital space is steeper than the scaling commonly reported for conventional VMC implementations with the number of electrons. Because these scaling estimates are expressed in terms of different variables, they do not provide a direct quantitative comparison of computational cost. Nevertheless, VMC avoids the explicit treatment of large virtual-orbital spaces and may therefore offer practical computational advantages for calculations involving larger basis sets and systems. The accuracy achieved by VMC, particularly for ionizations from more internal orbitals such as $1a_1$ , is attributed to the use of explicit electron-correlation functions. It is also evident that the DZ and HFL basis sets, when combined with explicit correlation, enable VMC to produce results comparable to those of higher-level methods. In contrast, the SZ basis set is less suitable for describing ionization of weakly bound electrons, yielding absolute (relative) errors of 0.68 eV (3.67%), 0.51 eV (3.46%), and 0.58 eV (4.60%) for the $1b_2$,$3a_1$ , and $1b_1$ orbitals, respectively.

VMC simulations without polarization functions were also carried out with the HFL basis set to assess the contribution of these terms to the description of ionization energies. Table 5 reports the results obtained with the HFL basis without polarization functions (HFL-NOP). The mean absolute deviation increases relative to the simulations including polarization. A closer inspection shows that, for ionizations from the ${1\mathrm{b}}_{1}$, ${3\mathrm{a}}_{1}$, and ${1\mathrm{b}}_{2}$ orbitals, the absolute deviations increase to 0.14, 0.46, and 0.73 eV, corresponding to relative errors of 1.11%, 3.12%, and 3.94%, respectively. These values are larger than those obtained with polarization functions, for which the errors do not exceed 0.30 eV (below 1.5%). By contrast, for the more internal orbitals (${1\mathrm{a}}_{1}$ and ${2\mathrm{a}}_{1}$), the deviations remain small even without polarization, at 0.20 and 1.74 eV, close to the values obtained in the polarized calculations. These results indicate that VMC/HFL-NOP provides reliable estimates for inner-shell ionizations, whereas polarization functions are essential to improve the description of valence-shell orbitals.

**Table 5:** Single ionization energies (eV) of H_2_O obtained with VMC using the HFL Slater-type basis set without polarization functions (HFL-NOP). AE denotes the absolute error with respect to experiment. MAE denotes the mean absolute error

Ionization	VMC-HFL-NOP	AE	Experiment [56]
1a_1_	539.497	0.20	539.70
2a_1_	34.35	1.74	32.61
1b_2_	19.24	0.73	18.51
3a_1_	14.28	0.46	14.74
1b_1_	12.48	0.14	12.62
MAE		0.65	

Table 6 presents the ionization energies of carbon monoxide (CO), comparing experimental values [57] with VMC results obtained with SZ, DZ, and HFL basis sets (all with polarization functions), as well as literature data for the CCSD [13] and CASSCF [58] methods. For the core orbitals (1σ and 2σ), the SZ basis set yields results similar to those obtained with the larger bases, with absolute deviations of only 2.13 and 1.07 eV, respectively. The HFL basis set gives slightly smaller errors (0.74 and 2.03 eV), whereas the DZ basis produces larger deviations (0.98 and 2.42 eV). The largest discrepancy in relative terms for the DZ and HFL calculations occurs for ionization of the 3σ orbital, with errors of 3.13% and 2.38%, respectively. As discussed above for the ${2\mathrm{a}}_{1}$ orbital of water, this result can be attributed to the intermediate character of 3σ, which lies in the inner-valence region and therefore requires a more refined treatment of electron correlation.

**Table 6:** Single ionization energies (eV) of CO obtained with VMC using the SZ, DZ and HFL basis sets (all with polarization functions). Values in parentheses are the absolute errors with respect to experiment. MAE denotes the mean absolute error

Ionization	VMC-SZ	VMC-DZ	VMC-HFL	Literature	Experiment [57]
1σ	544.18 (2.13)	543.03 (0.98)	542.79 (0.74)	542.61 (0.56)^a^	542.05
2σ	296.86 (1.07)	298.22 (2.42)	297.82 (2.03)	296.24 (0.49)^a^	295.79
3σ	36.77 (1.46)	39.43 (1.20)	39.14 (0.91)	––	38.23
4σ	20.32 (0.32)	20.43 (0.43)	19.80 (0.20)	19.80 (0.20)^b^	20
1π	16.86 (0.28)	17.35 (0.21)	17.22 (0.07)	17.10 (0.04)^b^	17.14
5σ	14.00 (0.44)	14.62 (0.17)	14.44 (0.05)	14.19 (0.23)^b^	14.44
MAE	0.95	0.91	0.67	(0.53^a^|0.22^b^)	

References: ^a^[58] and ^b^[13]

Using the DZ basis set, VMC yields a MAE of 0.91 eV and, although the largest absolute errors are associated with higher-energy ionizations, the corresponding relative errors tend to be less pronounced. For the outer-valence orbitals (1π and 5σ), the CCSD/aug-cc-pVTZ method is more accurate, with deviations below 0.05 eV [13]. Even so, the results obtained with the DZ and, in particular, the HFL basis sets are quite promising given the simplicity of VMC compared with more sophisticated approaches such as CCSD and CASSCF, both used with the aug-cc-pVTZ basis set.

Table 7 reports the ionization energies of CO obtained with the VMC approach using the HFL basis set without polarization functions (HFL-NOP). The results show excellent agreement with the experimental data, with absolute deviations ranging from 0.02 eV (1π) to 1.79 eV (2σ), and a mean absolute deviation of 0.67 eV.

**Table 7:** Single ionization energies (eV) of CO obtained with VMC using the HFL Slater-type basis set without polarization functions (HFL-NOP). AE denotes the absolute error with respect to experiment. MAE denotes the mean absolute error

Ionization	VMC-HFL-NOP	AE	Experiment [57]
1σ	542.76	0.72	542.05
2σ	297.57	1.79	295.79
3σ	39.43	1.17	38.23
4σ	20.20	0.21	20
1π	17.16	0.02	17.143
5σ	14.34	0.11	14.442
MAE		0.67	

Although omitting polarization functions typically reduces accuracy, the present results indicate that, for CO, the HFL-NOP basis set still provides a good description of the ionized electronic states, particularly for the more internal orbitals such as 1σ (error of 0.72 eV) and 2σ (error of 1.79 eV). Even for valence orbitals, such as 1π and 5σ, the deviations remain within acceptable limits (0.02 and 0.11 eV, respectively), suggesting that the absence of polarization functions does not significantly degrade the quality of the results.

These findings support the use of simpler basis sets in VMC calculations, which may reduce computational cost without a meaningful loss in accuracy for the systems considered. The excellent performance of VMC/HFL-NOP for CO, together with the results discussed above for water, further reinforces the viability of this approach even under less elaborate basis-set conditions.

## Conclusion

The present work provides a systematic assessment of Variational Monte Carlo (VMC) with a Boys–Handy explicit correlation factor for computing single valence and core ionization energies of atoms and small molecules. Slater-type basis sets (SZ, DZ, and HFL) were examined, both with and without polarization functions, to clarify the role of basis-set flexibility and explicit correlation in this context.

For the atomic systems, the overall trends are clear: SZ leads to noticeably larger deviations, particularly for inner-shell ionizations, whereas DZ and HFL yield consistently improved agreement with experiment across the ionization manifold considered. Importantly, the good performance obtained with DZ and HFL is achieved without fitting to experimental ionization energies, highlighting the predictive character of the variationally optimized explicit correlation wave function.

For the molecular cases, VMC with the HFL basis set performs competitively with higher-level approaches while employing a comparatively simple trial wave function and compact Slater-type basis sets. For H_2_O, VMC/HFL reaches a mean absolute error (MAE) of 0.42 eV, close to the 0.39 eV obtained with CCSD-GF. For CO, the corresponding VMC/HFL MAE is 0.67 eV. These results support the use of VMC as a practical route to ionization energies, while also indicating that orbitals with mixed inner-valence character remain the most challenging cases.

The impact of polarization functions depends on the ionized region. Removing polarization (HFL-NOP) generally degrades the description of valence ionizations, whereas inner-shell ionizations are affected far less. For H_2_O, the MAE with HFL-NOP is 0.65 eV, with good agreement retained for the ${1a}_{1}$ ionization. For CO, HFL-NOP yields a MAE of 0.67 eV, and remains particularly reliable for the 1σ core ionization.

Finally, variational optimization of the explicit correlation parameters is essential to the accuracy observed here, even though it increases computational effort. Taken together, the simplicity of VMC, the effectiveness of compact Slater-type basis sets, and the gains provided by explicit correlation make this approach attractive for extending ionization-energy calculations to larger systems. Future improvements may include explicit treatments of multiconfigurational (static) correlation and relativistic effects, particularly for challenging inner-valence and deep core ionizations.

## Supplementary Information

Below is the link to the electronic supplementary material.

ESM 1 (DOCX 242 KB)

## Data Availability

The optimized parameters of the explicit-correlation function for the atoms, ions, molecules, and molecular ions investigated in this work are available as Supplementary Material. The computer programs used to perform the Variational Monte Carlo calculations and parameter optimizations were developed by the authors and are available from the corresponding author upon reasonable request.

## References

[1] Lemierre V, Chrostowska A, Dargelos A, Chermette H (2005) Calculation of ionization potentials of small molecules: a comparative study of different methods. J Phys Chem A 109:8348–8355. 10.1021/jp050254c 16834226

[2] Ranasinghe DS, Margraf JT, Perera A, Bartlett RJ (2019) Vertical valence ionization potential benchmarks from equation-of-motion coupled cluster theory and QTP functionals. J Chem Phys 150:074108. 10.1063/1.5084728 30795671

[3] Custodio R (1985) The virial theorem as an entail for Koopmans’ theorem in atomic calculations. Theor Chim Acta 67:307–313. 10.1007/BF00529302

[4] Angeli C (1998) Physical interpretation of Koopmans’ theorem: a criticism of the current didactic presentation. J Chem Educ 75:1494. 10.1021/ed075p1494

[5] Koopmans T (1934) Über die Zuordnung von Wellenfunktionen und Eigenwerten zu den Einzelnen Elektronen Eines Atoms. Physica 1:104–113. 10.1016/S0031-8914(34)90011-2

[6] Custodio R, Takahata Y (1987) A modification of Koopmans’ theorem by imposition of the virial theorem in molecules. Theor Chim Acta 72:197–205. 10.1007/BF00527662

[7] Clouthier CM, Grein F (2005) MRCI studies on the electronic spectrum of GeO+. Chem Phys 315:35–40. 10.1016/j.chemphys.2005.03.021

[8] Eland JHD, Hochlaf M, Linusson P, Andersson E, Hedin L, Feifel R (2010) Triple ionization spectra by coincidence measurements of double Auger decay: the case of OCS. J Chem Phys 132:014311. 10.1063/1.3274648 20078165

[9] Streit L, Machado FBC, Custodio R (2011) Double ionization energies of HCl, HBr, Cl2 and Br 2 molecules: an MRCI study. Chem Phys Lett 506:22–25. 10.1016/j.cplett.2011.02.047

[10] Matthews DA (2020) EOM-CC methods with approximate triple excitations applied to core excitation and ionization energies. Mol Phys 118:e1771448. 10.1080/00268976.2020.1771448

[11] Hirata S, Nooijen M, Bartlett RJ (2000) High-order determinantal equation-of-motion coupled-cluster calculations for ionized and electron-attached states. Chem Phys Lett 328:459–468. 10.1016/S0009-2614(00)00965-9

[12] Maley SM, Lief GR, Buck RM, Sydora OL, Yang Q, Bischof SM, Ess DH (2023) Density functional theory and CCSD (T) evaluation of ionization potentials, redox potentials, and bond energies related to zirconocene polymerization catalysts. J Comput Chem 44:506–515. 10.1002/jcc.26890 35662063

[13] Stanton JF, Gauss J (1999) A simple scheme for the direct calculation of ionization potentials with coupled-cluster theory that exploits established excitation energy methods. J Chem Phys 111:8785–8788. 10.1063/1.479673

[14] Ortiz JV (1992) Electron propagator calculations on the adiabatic electron binding energies of C3. J Chem Phys 97:7531–7536. 10.1063/1.463472

[15] McKechnie S, Booth GH, Cohen AJ, Cole JM (2015) On the accuracy of density functional theory and wave function methods for calculating vertical ionization energies. J Chem Phys 142:194114. 10.1063/1.4921037 26001454

[16] Corzo HH, Galano A, Dolgounitcheva O, Zakrzewski VG, Ortiz JV (2015) NR2 and P3+: accurate, efficient electron-propagator methods for calculating valence, vertical ionization energies of closed-shell molecules. J Phys Chem A 119:8813–8821. 10.1021/acs.jpca.5b00942 26226061

[17] Wang B, Ding Y, Tian X (2024) Benchmarking model chemistry composite calculations for vertical ionization potential of molecular systems. Chin Chem Lett. 10.1016/j.cclet.2024.109721

[18] Opoku E, Pawłowski F, Ortiz JV (2024) New-generation electron-propagator methods for molecular electron-binding energies. J Phys Chem A 128:1399–1416. 10.1021/acs.jpca.3c08455 38377355

[19] Ortiz JV (2022) Recent progress in electron-propagator, extended-Koopmans-theorem and self-consistent-field approaches to the interpretation and prediction of electron binding energies. Advances in quantum chemistry. Elsevier, pp 109–155

[20] Takahata Y, Chong DP (2003) DFT calculation of core-electron binding energies. J Electron Spectrosc Relat Phenom 133:69–76. 10.1016/j.elspec.2003.08.001

[21] Delfino Angelotti WF, Streit L, Luiz Da Fonseca A, Custodio R (2008) Koopmans’ approximation applied in atoms and diatomic molecules using the diffusion quantum Monte Carlo method. Int J Quantum Chem 108:2459–2466. 10.1002/qua.21667

[22] Streit L, Custodio R (2009) The Auger spectra and the calculation of double-ionization potentials for H2O and NH3 using the diffusion quantum Monte Carlo method. Chem Phys Lett 482:148–152. 10.1016/j.cplett.2009.09.084

[23] Moskowitz JW, Kalos MH (1981) A new look at correlations in atomic and molecular systems. I. Application of fermion monte carlo variational method. Int J Quantum Chem 20:1107–1119. 10.1002/qua.560200508

[24] Schmidt KE, Moskowitz JW (1990) Correlated Monte Carlo wave functions for the atoms He through Ne. J Chem Phys 93:4172–4178. 10.1063/1.458750

[25] Moskowitz JW, Schmidt KE (1992) Correlated Monte Carlo wave functions for some cations and anions of the first row atoms. J Chem Phys 97:3382–3385. 10.1063/1.463938

[26] Galek PTA, Handy NC, Lester WA (2006) Quantum Monte Carlo studies on small molecules. Mol Phys 104:3069–3085. 10.1080/00268970600962071

[27] Drummond ND, López Ríos P, Ma A, Trail JR, Spink GG, Towler MD, Needs RJ (2006) Quantum Monte Carlo study of the Ne atom and the Ne+ ion. J Chem Phys 124:224104. 10.1063/1.2204600 16784260

[28] Maldonado P, Sarsa A, Buendía E, Gálvez FJ (2010) Quantum Monte Carlo ground state energies for the singly charged ions from Li through Ar. J Chem Phys 133:064102. 10.1063/1.3467879 20707556

[29] Seth P, Ríos PL, Needs RJ (2011) Quantum Monte Carlo study of the first-row atoms and ions. J Chem Phys 134:084105. 10.1063/1.3554625 21361525

[30] Sabzevari I, Mahajan A, Sharma S (2020) An accelerated linear method for optimizing non-linear wavefunctions in variational Monte Carlo. J Chem Phys 152:024111. 10.1063/1.5125803 31941334

[31] Cuzzocrea A, Scemama A, Briels WJ, Moroni S, Filippi C (2020) Variational principles in quantum Monte Carlo: the troubled story of variance minimization. J Chem Theory Comput 16:4203–4212. 10.1021/acs.jctc.0c00147 PMC7365558 32419451

[32] Otis L, Neuscamman E (2023) Optimization stability in excited-state-specific variational Monte Carlo. J Chem Theory Comput 19:767–782. 10.1021/acs.jctc.2c00642 36662538

[33] Trofimov AB, Schirmer J (2005) Molecular ionization energies and ground- and ionic-state properties using a non-Dyson electron propagator approach. J Chem Phys 123:144115. 10.1063/1.2047550 16238382

[34] Banerjee S, Sokolov AYu (2019) Third-order algebraic diagrammatic construction theory for electron attachment and ionization energies: conventional and Green’s function implementation. J Chem Phys 151:224112. 10.1063/1.5131771 31837698

[35] Mussard B, Coccia E, Assaraf R, Otten M, Umrigar CJ, Toulouse J (2018) Time-dependent linear-response variational Monte Carlo. Advances in quantum chemistry. Elsevier, pp 255–270

[36] Foulkes WMC, Mitas L, Needs RJ, Rajagopal G (2001) Quantum Monte Carlo simulations of solids. Rev Mod Phys 73:33–83. 10.1103/RevModPhys.73.33

[37] Hammond BL, Lester WA, Reynolds PJ (1994) Monte Carlo methods in ab initio quantum chemistry. World Scientific, Singapore ; River Edge, NJ

[38] Metropolis N, Rosenbluth AW, Rosenbluth MN, Teller AH, Teller E (1953) Equation of state calculations by fast computing machines. J Chem Phys 21:1087–1092. 10.1063/1.1699114 41342507

[39] Angelotti WFD, Politi JRS, Custodio R (2023) Preliminary assessment of the first-order density matrix in quantum Monte Carlo from density matrix theory. J Chem Theory Comput 19:3861–3867. 10.1021/acs.jctc.2c01174 37294855

[40] Dos Santos Politi JR, Custodio R (2003) Comments on the quantum Monte Carlo method and the density matrix theory. J Chem Phys 118:4781–4783. 10.1063/1.1558393

[41] Kussmann J, Ochsenfeld C (2008) Linear-scaling fixed-node diffusion quantum Monte Carlo: accounting for the nodal information in a density matrix-based scheme. J Chem Phys 128:134104. 10.1063/1.2884920 18397050

[42] Kussmann J, Riede H, Ochsenfeld C (2007) Density matrix-based variational quantum Monte Carlo providing an asymptotically linear scaling behavior for the local energy. Phys Rev B 75:165107. 10.1103/PhysRevB.75.165107

[43] Clementi E, Roetti C (1974) Roothaan-Hartree-Fock atomic wavefunctions. At Data Nucl Data Tables 14:177–478. 10.1016/S0092-640X(74)80016-1

[44] Koga T, Thakkar AJ (1993) Optimal single-zeta description for the atoms Al through Xe. Theor Chim Acta 85:363–370. 10.1007/BF01113429

[45] Koga T, Seki Y, Thakkar AJ (1993) Improved double-zeta description for the atoms Li through Xe. Bull Chem Soc Jpn 66:3135–3141. 10.1246/bcsj.66.3135

[46] Koga T, Tatewaki H, Thakkar AJ (1993) Roothaan-Hartree-Fock wave functions for atoms with *Z* ≤54. Phys Rev A 47:4510–4512. 10.1103/PhysRevA.47.4510 9909462

[47] Dempster AP, Laird NM, Rubin DB (1977) Maximum likelihood from incomplete data via the *EM* algorithm. J R Stat Soc Ser B Stat Methodol 39:1–22. 10.1111/j.2517-6161.1977.tb01600.x

[48] Nelder JA, Mead R (1965) A simplex method for function minimization. Comput J 7:308–313. 10.1093/comjnl/7.4.308

[49] Frisch MJ, Trucks GW, Schlegel HB, Scuseria GE, Robb MA, Cheeseman JR, Scalmani G, Barone V, Mennucci B, Petersson GA, Nakatsuji H, Caricato M, Li X, Hratchian HP, Izmaylov AF, Bloino J, Zheng G, Sonnenberg JL, Hada M, Ehara M, Toyota K, Fukuda R, Hasegawa J, Ishida M, Nakajima T, Honda Y, Kitao O, Nakai H, Vreven T, Montgomery JA Jr, Peralta JE, Ogliaro F, Bearpark M, Heyd JJ, Brothers E, Kudin KN, Staroverov VN, Kobayashi R, Normand J, Raghavachari K, Rendell A, Burant JC, Iyengar SS, Tomasi J, Cossi M, Rega N, Millam JM, Klene M, Knox JE, Cross JB, Bakken V, Adamo C, Jaramillo J, Gomperts R, Stratmann RE, Yazyev O, Austin AJ, Cammi R, Pomelli C, Ochterski JW, Martin RL, Morokuma K, Zakrzewski VG, Voth GA, Salvador P, Dannenberg JJ, Dapprich S, Daniels AD, Farkas Ö, Foresman JB, Ortiz JV, Cioslowski J, Fox DJ (2009) Gaussian 09 Revision D.01

[50] Chisholm CDH, Dalgarno A, Innes FR (1969) Tables of one- and two-particle coefficients of fractional parentage for configurations SλS′μpq. Advances in atomic and molecular physics. Elsevier, pp 297–335

[51] Shirley DA, Martin RL, Kowalczyk SP, McFeely FR, Ley L (1977) Core-electron binding energies of the first thirty elements. Phys Rev B 15:544–552. 10.1103/PhysRevB.15.544

[52] Bearden JA, Burr AF (1967) Reevaluation of X-ray atomic energy levels. Rev Mod Phys 39:125–142. 10.1103/RevModPhys.39.125

[53] Cardona M, Ley L (1978) Photoemission in solids I. Springer, Berlin Heidelberg, Berlin, Heidelberg

[54] Kramida A, Ralchenko Y (2023) NIST Atomic Spectra Database, NIST Standard Reference Database 78. 10.18434/T4W30F

[55] Nooijen M, Snijders JG (1995) Second order many-body perturbation approximations to the coupled cluster Green’s function. J Chem Phys 102:1681–1688. 10.1063/1.468900

[56] Kimura K (1981) Handbook of He I photoelectron spectra of fundamental organic molecules: ionization energies, ab initio assignments and valence electronic structure for 200 molecules. Japan Scient. Soc. Pr, Tokyo

[57] Siegbahn K (1969) ESCA applied to free molecules. North-Holland Pub. Co, Amsterdam

[58] Tashiro M, Ehara M, Fukuzawa H, Ueda K, Buth C, Kryzhevoi NV, Cederbaum LS (2010) Molecular double core hole electron spectroscopy for chemical analysis. J Chem Phys 132:184302. 10.1063/1.3408251

