# Supplementary material for: Valence and core ionization energies of atoms and small molecules using variational Monte Carlo with explicit correlation and Slater-type basis sets
Source: J Mol Model. 2026 Oct 9;32(11):408. doi: 10.1007/s00894-026-06987-y (PMC13652765; doi:10.1007/s00894-026-06987-y)
Supplement: Supplementary file 1 [file 894_2026_6987_MOESM1_ESM.docx]

***Supplementary Material***

**Valence and Core Ionization Energies of Atoms and Small Molecules Using Variational Monte Carlo with Explicit Correlation and Slater-Type Basis Sets**

*Josué de Jesus Oliveira Araujo, Pedro Oliveira Mariz de Carvalho and Rogério Custodio*^*^

*Instituto de Química*

*Universidade Estadual de Campinas*

*Barão Geraldo*

*13083-970 Campinas – São Paulo, Brasil, P. O. Box 6154*

Index

[**Table S.1.** Optimized parameters for the coefficients of the explicit Boys–Handy correlation function for the elements from He to F using SZ basis set. Values in parenthesis are the charge and multiplicity.	4](#_Toc230735125)

[**Table S.2.** Optimized parameters for the coefficients of the explicit Boys–Handy correlation function for the elements from Ne to Si using SZ basis set. Values in parenthesis are the charge and multiplicity.	5](#_Toc230735126)

[**Table S.3.** Optimized parameters for the coefficients of the explicit Boys–Handy correlation function for the elements from P to Ar using SZ basis set. Values in parenthesis are the charge and multiplicity.	6](#_Toc230735127)

[**Table S.4.** Optimized parameters for the coefficients of the explicit Boys–Handy correlation function for the elements from He to F using DZ basis set. Values in parenthesis are the charge and multiplicity.	7](#_Toc230735128)

[**Table S.5.** Optimized parameters for the coefficients of the explicit Boys–Handy correlation function for the elements from Ne to Si using DZ basis set. Values in parenthesis are the charge and multiplicity.	8](#_Toc230735129)

[**Table S.6.** Optimized parameters for the coefficients of the explicit Boys–Handy correlation function for the elements from P to Ar using DZ basis set. Values in parenthesis are the charge and multiplicity.	9](#_Toc230735130)

[**Table S.7.** Optimized parameters for the coefficients of the explicit Boys–Handy correlation function for the elements from He to F using NHF basis set. Values in parenthesis are the charge and multiplicity.	10](#_Toc230735131)

[**Table S.8.** Optimized parameters for the coefficients of the explicit Boys–Handy correlation function for the elements from Ne to Si using NHF basis set. Values in parenthesis are the charge and multiplicity.	11](#_Toc230735132)

[**Table S.9.** Optimized parameters for the coefficients of the explicit Boys–Handy correlation function for the elements from P to Ar using NHF basis set. Values in parenthesis are the charge and multiplicity.	12](#_Toc230735133)

[**Table S.10.** Calculated energies (a.u.) for the atoms and ions from He to F using SZ, DZ and NHF basis sets.	13](#_Toc230735134)

[**Table S.11.** Calculated energies (a.u.) for the atoms and ions from Ne to Si using SZ, DZ and NHF basis sets.	14](#_Toc230735135)

[**Table S.12.** Calculated energies (a.u.) for the atoms and ions from P to Ar using SZ, DZ and NHF basis sets.	15](#_Toc230735136)

[**Table S.13.** Optimized parameters for the coefficients of the explicit Boys–Handy correlation function for H_2_O and CO using SZ basis set. Values in parenthesis are the charge and multiplicity.	16](#_Toc230735137)

[**Table S.14.** Optimized parameters for the coefficients of the explicit Boys–Handy correlation function for H_2_O and CO using DZ basis set. Values in parenthesis are the charge and multiplicity.	17](#_Toc230735138)

[**Table S.15.** Optimized parameters for the coefficients of the explicit Boys–Handy correlation function for H_2_O and CO using NHF basis set. Values in parenthesis are the charge and multiplicity.	18](#_Toc230735139)

[**Table S.16.** Optimized parameters for the coefficients of the explicit Boys–Handy correlation function for H_2_O and CO using NHF_SP basis set. Values in parenthesis are the charge and multiplicity.	19](#_Toc230735140)

[**Table S.17.** Calculated energies for H_2_O and CO using SZ, DZ, NHF and NHF_SP basis sets.	20](#_Toc230735141)

### **Table S.1.** Optimized parameters for the coefficients of the explicit Boys–Handy correlation function for the elements from He to F using SZ basis set. Values in parenthesis are the charge and multiplicity.

| Atom | System | $c_{1A}$ | $c_{2A}$ | $c_{3A}$ | $c_{4A}$ | $c_{5A}$ | $c_{6A}$ | $c_{7A}$ | $c_{8A}$ | $c_{9A}$ |
| --- | --- | --- | --- | --- | --- | --- | --- | --- | --- | --- |
| He | (0,1) 1s² | 0.5 | 0.21657 | -0.55047 | 0.37718 | -0.98333 | 1.14906 | 0.25824 | -2.63378 | 0.91192 |
| Li | (0,2) 2s¹ | 0.5 | -0.13287 | 1.11363 | -1.41532 | -1.14657 | 3.38228 | -2.67036 | -0.89610 | 0.48477 |
|  | (+1,1) 1s¹2s¹ | 0.5 | -1.17119 | 4.32493 | -3.91587 | -2.67817 | 9.08513 | -10.18220 | -1.52292 | 0.54435 |
|  | (+1,3) 1s¹2s¹ | 0.5 | -3.44399 | 9.08308 | -6.58142 | -2.94714 | 10.33323 | -11.63407 | -1.27048 | 0.29310 |
|  | (+1,1) 1s² | 0.5 | -0.19920 | 0.66319 | -0.94200 | -2.05986 | 5.83529 | -4.58604 | -3.09907 | 1.01466 |
| Be | (0,1) 2s² | 0.5 | 0.65182 | -2.50101 | 0.78743 | -1.02243 | 3.79035 | -4.18024 | -2.22057 | 2.15985 |
|  | (+1,2) 1s¹2s² | 0.5 | 0.66491 | -2.53626 | 0.84460 | -1.36414 | 6.18093 | -7.79699 | -3.29738 | 2.28108 |
|  | (+1,2) 2s¹ | 0.5 | 0.04646 | 0.12151 | -0.60569 | -1.47877 | 5.14565 | -5.04854 | -0.62062 | 0.35745 |
| B | (0,2) 2p¹⁰⁰ | 0.5 | 0.78091 | -3.50363 | 1.89082 | -0.67521 | 2.42256 | -3.10070 | -1.87034 | 2.02360 |
|  | (+1,1) 1s2p¹⁰⁰ | 0.5 | 0.24126 | -1.74231 | 0.61944 | -0.62004 | 2.47786 | -3.96358 | -2.36669 | 1.88929 |
|  | (+1,3) 1s2p¹⁰⁰ | 0.5 | 0.23125 | -1.47156 | 0.44362 | -0.59233 | 2.09451 | -3.56011 | -2.06348 | 1.71212 |
|  | (+1,1) 2s¹2p¹⁰⁰ | 0.5 | 0.69216 | -3.25833 | 1.71237 | -1.03623 | 3.59032 | -4.60263 | -1.69542 | 2.12189 |
|  | (+1,3) 2s¹2p¹⁰⁰ | 0.5 | 0.59492 | -2.57027 | 1.75779 | -0.95638 | 3.06577 | -3.51393 | -0.30227 | 0.64299 |
|  | (+1,1) 2s² | 0.5 | 0.51452 | -2.59600 | 1.08640 | -1.02826 | 4.28894 | -5.33256 | -2.23873 | 2.16897 |
| C | (0,3) 2p¹¹⁰ | 0.5 | 0.52561 | -3.05502 | 1.79232 | -0.32104 | 1.06253 | -1.83509 | -1.77839 | 1.85207 |
|  | (+1,2) 1s¹2p¹¹⁰ | 0.5 | -0.31329 | -0.42747 | -0.18828 | -0.19437 | 0.49019 | -1.73809 | -2.15935 | 1.70128 |
|  | (+1,4) 1s¹2p¹¹⁰ | 0.5 | -0.34841 | 0.03121 | -0.52573 | -0.16191 | -0.02247 | -1.14623 | -1.75180 | 1.44621 |
|  | (+1,2) 2s¹2p¹¹⁰ | 0.5 | 0.48130 | -3.12760 | 1.92148 | -0.58359 | 1.76056 | -2.95648 | -1.68496 | 2.14601 |
|  | (+1,4) 2s¹2p¹¹⁰ | 0.5 | 0.41543 | -2.66032 | 1.91141 | -0.50672 | 1.36598 | -2.09038 | -0.65811 | 1.00301 |
|  | (+1,2) 2p¹⁰⁰ | 0.5 | 0.41211 | -2.68332 | 1.45593 | -0.59040 | 2.19956 | -3.32481 | -1.76353 | 1.96872 |
| N | (0,4) 2p¹¹¹ | 0.5 | 0.09561 | -1.86938 | 1.03455 | -0.04578 | -0.04091 | -0.64209 | -1.78224 | 1.64211 |
|  | (+1,3) 1s¹2p¹¹¹ | 0.5 | -0.79959 | 0.89441 | -1.10767 | -0.00169 | -0.53479 | -0.38275 | -2.06562 | 1.52141 |
|  | (+1,5) 1s¹2p¹¹¹ | 0.5 | -0.74944 | 1.16684 | -1.31198 | 0.01777 | -0.99943 | 0.22367 | -1.64937 | 1.21321 |
|  | (+1,3) 2s¹2p¹¹¹ | 0.5 | 0.02976 | -1.90432 | 1.19583 | -0.24012 | 0.34592 | -1.43579 | -1.70109 | 1.94481 |
|  | (+1,5) 2s¹2p¹¹¹ | 0.5 | -0.07240 | -1.42588 | 0.96926 | -0.16159 | 0.01800 | -0.71299 | -1.01379 | 1.12986 |
|  | (+1,3) 2p¹¹⁰ | 0.5 | 0.05020 | -1.73183 | 0.87883 | -0.22894 | 0.60236 | -1.61134 | -1.67846 | 1.76185 |
| O | (0,3) 2p²¹¹ | 0.5 | -0.29883 | -0.67984 | 0.00973 | 0.11977 | -0.79601 | 0.23597 | -2.03855 | 1.72124 |
|  | (+1,2) 1s¹2p²¹¹ | 0.5 | -1.06602 | 1.80120 | -2.04443 | 0.05215 | -1.07851 | 0.43884 | -2.22920 | 1.67039 |
|  | (+1,4) 1s¹2p²¹¹ | 0.5 | -0.99221 | 1.81103 | -2.04393 | 0.05988 | -1.30802 | 0.76955 | -1.99568 | 1.48069 |
|  | (+1,2) 2s¹2p²¹¹ | 0.5 | -0.49399 | -0.16594 | -0.25785 | -0.00336 | -0.76972 | -0.08517 | -1.82656 | 1.85788 |
|  | (+1,4) 2s¹2p²¹¹ | 0.5 | -0.57370 | 0.17087 | -0.61816 | 0.03201 | -0.93111 | 0.22411 | -1.68105 | 1.67909 |
|  | (+1,2) 2p¹¹¹ | 0.5 | -0.36143 | -0.50532 | -0.13108 | 0.00292 | -0.46604 | -0.36821 | -1.86679 | 1.76173 |
|  | (+1,4) 2p¹¹¹ | 0.5 | -0.36973 | -0.47423 | -0.01844 | 0.00832 | -0.49332 | -0.26914 | -1.67122 | 1.53149 |
|  | (+1,2) 2p²¹⁰ | 0.5 | -0.36146 | -0.52153 | -0.19479 | 0.00288 | -0.44585 | -0.43451 | -2.01743 | 1.91461 |
| F | (0,2) 2p²²¹ | 0.5 | -0.66438 | 0.48041 | -0.97350 | 0.19100 | -1.23541 | 0.87676 | -2.15716 | 1.69519 |
|  | (+1,1) 1s2p²²¹ | 0.5 | -1.21314 | 2.34071 | -2.58962 | 0.03135 | -1.25266 | 0.91242 | -2.29254 | 1.67525 |
|  | (+1,3) 1s2p²²¹ | 0.5 | -1.17477 | 2.32242 | -2.57795 | 0.02665 | -1.32382 | 1.03587 | -2.18928 | 1.59536 |
|  | (+1,1) 2s2p²²¹ | 0.5 | -0.91047 | 1.28147 | -1.50573 | 0.09806 | -1.39087 | 0.85455 | -1.87849 | 1.73492 |
|  | (+1,3) 2s2p²²¹ | 0.5 | -0.96541 | 1.49782 | -1.74343 | 0.11250 | -1.45508 | 0.97586 | -1.83708 | 1.68513 |
|  | (+1,1) 2p²¹¹ | 0.5 | -0.74769 | 0.72446 | -1.20135 | 0.11405 | -1.08969 | 0.49711 | -2.03986 | 1.76866 |
|  | (+1,3) 2p²¹¹ | 0.5 | -0.74962 | 0.72559 | -1.15761 | 0.11963 | -1.11244 | 0.54077 | -1.98751 | 1.70086 |
|  | (+1,1) 2p²²⁰ | 0.5 | -0.73160 | 0.66759 | -1.21781 | 0.11193 | -1.07386 | 0.44758 | -2.15011 | 1.88683 |

### **Table S.2.** Optimized parameters for the coefficients of the explicit Boys–Handy correlation function for the elements from Ne to Si using SZ basis set. Values in parenthesis are the charge and multiplicity.

| Atom | System | $c_{1A}$ | $c_{2A}$ | $c_{3A}$ | $c_{4A}$ | $c_{5A}$ | $c_{6A}$ | $c_{7A}$ | $c_{8A}$ | $c_{9A}$ |
| --- | --- | --- | --- | --- | --- | --- | --- | --- | --- | --- |
| Ne | (0,1) 2p²²² | 0.5 | -0.93030 | 1.36344 | -1.72491 | 0.19725 | -1.44233 | 1.32851 | -2.20091 | 1.61767 |
|  | (+1,2) 1s2p²²² | 0.5 | -1.31826 | 2.73811 | -2.99251 | -0.03430 | -1.21546 | 1.17417 | -2.31074 | 1.63259 |
|  | (+1,2) 2s2p²²² | 0.5 | -1.22706 | 2.41608 | -2.51886 | 0.11786 | -1.66691 | 1.44699 | -1.89963 | 1.61563 |
|  | (+1,2) 2p²²¹ | 0.5 | -1.04490 | 1.70717 | -2.06669 | 0.14631 | -1.42133 | 1.10509 | -2.12351 | 1.72583 |
| Na | (0,2) 3s¹ | 0.5 | -1.54983 | 3.51597 | -3.64985 | 0.06268 | -1.11115 | 1.14376 | -1.89926 | 1.60175 |
|  | (+1,1) 1s¹3s¹ | 0.5 | -2.08777 | 5.43872 | -5.35890 | -0.31081 | -0.35294 | 0.36775 | -1.77711 | 1.59214 |
|  | (+1,3) 1s¹3s¹ | 0.5 | -2.13417 | 5.59803 | -5.49337 | -0.31860 | -0.32926 | 0.33636 | -1.76647 | 1.59238 |
|  | (+1,1) 2s¹3s¹ | 0.5 | -2.22716 | 5.94752 | -5.64856 | -0.13734 | -1.04986 | 0.95928 | -1.25605 | 1.54566 |
|  | (+1,3) 2s¹3s¹ | 0.5 | -2.22142 | 5.94371 | -5.65872 | -0.13809 | -1.06053 | 0.97019 | -1.23266 | 1.54275 |
|  | (+1,1) 2p²²¹3s¹ | 0.5 | -2.03266 | 5.20499 | -5.13296 | -0.08814 | -0.91594 | 0.71405 | -1.40030 | 1.60791 |
|  | (+1,3) 2p²²¹3s¹ | 0.5 | -2.06451 | 5.31005 | -5.22153 | -0.09288 | -0.89608 | 0.69067 | -1.39502 | 1.60640 |
|  | (+1,1) 2p²²² | 0.5 | -1.57506 | 3.60424 | -3.83610 | 0.07371 | -1.27543 | 1.30360 | -2.08684 | 1.70563 |
| Mg | (0,1) 3s² | 0.5 | -1.73192 | 4.08720 | -4.07574 | -0.16566 | -0.32570 | 0.24485 | -1.59267 | 1.61710 |
|  | (+1,2) 1s¹3s² | 0.5 | -1.78258 | 4.33117 | -4.27174 | -0.48898 | 0.58654 | -0.75112 | -1.68095 | 1.57082 |
|  | (+1,2) 2s¹3s² | 0.5 | -2.08174 | 5.35585 | -4.98887 | -0.33444 | -0.06478 | -0.17474 | -1.14936 | 1.48329 |
|  | (+1,2) 2p⁵3s² | 0.5 | -1.90353 | 4.62020 | -4.45299 | -0.26504 | -0.01444 | -0.36456 | -1.33776 | 1.57683 |
|  | (+1,2) 2s3s | 0.5 | -1.85860 | 4.67067 | -4.69235 | -0.11775 | -0.67038 | 0.73399 | -1.58086 | 1.53878 |
| Al | (0,2) 3p¹⁰⁰ | 0.5 | -1.47572 | 3.11934 | -3.14223 | -0.23272 | 0.44188 | -0.70039 | -1.78880 | 1.54104 |
|  | (+1,1) 1s¹3p¹⁰⁰ | 0.5 | -1.39005 | 2.98430 | -3.02355 | -0.47800 | 0.95581 | -1.16894 | -1.73428 | 1.42004 |
|  | (+1,3) 1s¹3p¹⁰⁰ | 0.5 | -1.38557 | 2.96190 | -3.00672 | -0.47885 | 0.96900 | -1.18600 | -1.76361 | 1.43559 |
|  | (+1,1) 2s¹3p¹⁰⁰ | 0.5 | -1.71516 | 4.06016 | -3.77929 | -0.36323 | 0.54107 | -0.85248 | -1.34780 | 1.35041 |
|  | (+1,3) 2s¹3p¹⁰⁰ | 0.5 | -1.73315 | 4.11210 | -3.81369 | -0.36435 | 0.55646 | -0.88080 | -1.34593 | 1.34759 |
|  | (+1,1) 2p¹²²3p¹⁰⁰ | 0.5 | -1.59660 | 3.47763 | -3.42315 | -0.24333 | 0.43736 | -0.89124 | -1.75834 | 1.56719 |
|  | (+1,3) 2p¹²²3p¹⁰⁰ | 0.5 | -1.59472 | 3.48866 | -3.41463 | -0.27787 | 0.43808 | -0.87799 | -1.55889 | 1.52379 |
|  | (+1,1) 2p²¹²3p¹⁰⁰ | 0.5 | -1.58717 | 3.46008 | -3.37879 | -0.27279 | 0.43539 | -0.87053 | -1.57304 | 1.51239 |
|  | (+1,3) 2p²¹²3p¹⁰⁰ | 0.5 | -1.59428 | 3.48936 | -3.40364 | -0.27903 | 0.43920 | -0.87293 | -1.54391 | 1.50710 |
|  | (+1,1) 3s⁰3p¹⁰⁰ | 0.5 | -1.71516 | 3.95982 | -3.94116 | -0.23719 | 0.35537 | -0.64199 | -1.89806 | 1.62452 |
|  | (+1,3) 3s⁰3p¹⁰⁰ | 0.5 | -1.74370 | 4.17626 | -4.11810 | -0.22422 | 0.21632 | -0.36564 | -1.68491 | 1.42749 |
|  | (+1,1) 3s² | 0.5 | -1.71316 | 3.95508 | -3.90417 | -0.26682 | 0.43641 | -0.75092 | -1.76106 | 1.57395 |
| Si | (0,3) 3p¹¹⁰ | 0.5 | -1.37537 | 2.73490 | -2.75355 | -0.30436 | 0.70892 | -0.97897 | -1.60908 | 1.47266 |
|  | (+1,2) 1s¹3p¹¹⁰ | 0.5 | -1.24761 | 2.44620 | -2.51427 | -0.50502 | 1.16436 | -1.39236 | -1.64197 | 1.36617 |
|  | (+1,4) 1s¹3p¹¹⁰ | 0.5 | -1.25629 | 2.49593 | -2.55241 | -0.51461 | 1.16885 | -1.40099 | -1.59125 | 1.35159 |
|  | (+1,2) 2s¹3p¹¹⁰ | 0.5 | -1.52813 | 3.38881 | -3.14985 | -0.42404 | 0.85127 | -1.17070 | -1.23374 | 1.27207 |
|  | (+1,4) 2s¹3p¹¹⁰ | 0.5 | -1.53479 | 3.42595 | -3.15948 | -0.42311 | 0.83372 | -1.13218 | -1.17760 | 1.21978 |
|  | (+1,2) 2p¹²²3p¹¹⁰ | 0.5 | -1.49616 | 3.09530 | -3.01707 | -0.29037 | 0.65779 | -1.10555 | -1.62109 | 1.47052 |
|  | (+1,4) 2p¹²²3p¹¹⁰ | 0.5 | -1.47566 | 3.03839 | -2.94881 | -0.32247 | 0.66667 | -1.10153 | -1.43730 | 1.42413 |
|  | (+1,2) 2p²²¹3p¹¹⁰ | 0.5 | -1.47437 | 3.02928 | -2.94590 | -0.32018 | 0.66249 | -1.10095 | -1.45453 | 1.43723 |
|  | (+1,4) 2p²²¹3p¹¹⁰ | 0.5 | -1.45223 | 2.95803 | -2.87613 | -0.32016 | 0.66100 | -1.08952 | -1.44308 | 1.42086 |
|  | (+1,2) 3s¹3p¹¹⁰ | 0.5 | -1.55078 | 3.33640 | -3.33069 | -0.32955 | 0.72063 | -1.05844 | -1.71397 | 1.59483 |
|  | (+1,4) 3s¹3p¹¹⁰ | 0.5 | -1.55545 | 3.52616 | -3.40813 | -0.31200 | 0.54290 | -0.64724 | -1.33602 | 1.19815 |
|  | (+1,2) 3p¹⁰⁰ | 0.5 | -1.51322 | 3.23958 | -3.21631 | -0.32255 | 0.70906 | -1.01001 | -1.62459 | 1.47859 |

### **Table S.3.** Optimized parameters for the coefficients of the explicit Boys–Handy correlation function for the elements from P to Ar using SZ basis set. Values in parenthesis are the charge and multiplicity.

| Atom | System | $c_{1A}$ | $c_{2A}$ | $c_{3A}$ | $c_{4A}$ | $c_{5A}$ | $c_{6A}$ | $c_{7A}$ | $c_{8A}$ | $c_{9A}$ |
| --- | --- | --- | --- | --- | --- | --- | --- | --- | --- | --- |
| P | (0,4) 3p¹¹¹ | 0.5 | -1.75369 | 3.81115 | -3.51890 | -0.20085 | -0.15249 | -0.09080 | -1.69700 | 1.44032 |
|  | (+1,3) 1s¹3p¹¹¹ | 0.5 | -1.82112 | 4.06134 | -3.76188 | -0.40006 | 0.36514 | -0.55722 | -1.96126 | 1.45842 |
|  | (+1,5) 1s¹3p¹¹¹ | 0.5 | -1.81910 | 4.07807 | -3.75567 | -0.42028 | 0.38578 | -0.56268 | -1.86962 | 1.41321 |
|  | (+1,3) 2s¹3p¹¹¹ | 0.5 | -1.99992 | 4.92058 | -4.38501 | -0.25139 | -0.27565 | 0.04895 | -1.60970 | 1.25970 |
|  | (+1,5) 2s¹3p¹¹¹ | 0.5 | -2.00642 | 4.92418 | -4.32489 | -0.25458 | -0.26436 | 0.04244 | -1.53396 | 1.18859 |
|  | (+1,3) 2p¹²²3p¹¹¹ | 0.5 | -1.87590 | 4.23398 | -3.86538 | -0.17813 | -0.28663 | -0.08831 | -1.65674 | 1.40355 |
|  | (+1,5) 2p¹²²3p¹¹¹ | 0.5 | -1.91934 | 4.37963 | -3.97214 | -0.17928 | -0.27427 | -0.09639 | -1.64900 | 1.39000 |
|  | (+1,3) 3s¹3p¹¹¹ | 0.5 | -1.91158 | 4.31994 | -4.03600 | -0.23917 | -0.11573 | -0.25073 | -1.86302 | 1.64316 |
|  | (+1,5) 3s¹3p¹¹¹ | 0.5 | -1.95157 | 4.58807 | -4.22431 | -0.21970 | -0.25078 | 0.04968 | -1.61457 | 1.36046 |
|  | (+1,3) 3p¹¹⁰ | 0.5 | -1.86094 | 4.20914 | -3.92111 | -0.21229 | -0.22237 | -0.05367 | -1.73797 | 1.47967 |
| S | (0,3) 3p²¹¹ | 0.5 | -1.26890 | 2.24969 | -2.26152 | -0.34266 | 1.01330 | -1.33759 | -1.58615 | 1.42917 |
|  | (+1,2) 1s¹3p²¹¹ | 0.5 | -1.21294 | 2.21000 | -2.25680 | -0.51013 | 1.40083 | -1.69231 | -1.65653 | 1.36372 |
|  | (+1,4) 1s¹3p²¹¹ | 0.5 | -1.18280 | 2.11290 | -2.16275 | -0.51160 | 1.40922 | -1.70267 | -1.64891 | 1.35029 |
|  | (+1,2) 2s¹3p²¹¹ | 0.5 | -1.36578 | 2.73358 | -2.57399 | -0.44958 | 1.20374 | -1.57173 | -1.32269 | 1.27028 |
|  | (+1,4) 2s¹3p²¹¹ | 0.5 | -1.37555 | 2.77480 | -2.59139 | -0.44621 | 1.18629 | -1.53846 | -1.28912 | 1.23232 |
|  | (+1,2) 2p¹²²3p²¹¹ | 0.5 | -1.44433 | 2.83072 | -2.74206 | -0.31051 | 0.89314 | -1.35007 | -1.64721 | 1.43109 |
|  | (+1,4) 2p¹²²3p²¹¹ | 0.5 | -1.45800 | 2.88607 | -2.78101 | -0.30847 | 0.87976 | -1.32836 | -1.62599 | 1.40917 |
|  | (+1,2) 2p²¹²3p²¹¹ | 0.5 | -1.46221 | 2.89353 | -2.78731 | -0.30566 | 0.88138 | -1.33620 | -1.64792 | 1.42011 |
|  | (+1,4) 2p²¹²3p²¹¹ | 0.5 | -1.44455 | 2.83460 | -2.72123 | -0.32630 | 0.88521 | -1.33515 | -1.53434 | 1.39782 |
|  | (+1,2) 3s¹3p⁴ | 0.5 | -1.34141 | 2.46706 | -2.49384 | -0.39667 | 1.15658 | -1.60337 | -1.65712 | 1.57804 |
|  | (+1,2) 3s¹3p⁴ | 0.5 | -1.35292 | 2.60921 | -2.56728 | -0.38444 | 1.04635 | -1.35870 | -1.43556 | 1.34374 |
|  | (+1,2) 3p¹¹¹ | 0.5 | -1.40841 | 2.76683 | -2.72184 | -0.35665 | 1.02445 | -1.37235 | -1.59596 | 1.41427 |
|  | (+1,4) 3p¹¹¹ | 0.5 | -1.40124 | 2.75147 | -2.67685 | -0.35752 | 1.01817 | -1.34251 | -1.53980 | 1.35459 |
|  | (+1,2) 3p²¹⁰ | 0.5 | -1.38803 | 2.69214 | -2.67656 | -0.35725 | 1.03455 | -1.38999 | -1.63084 | 1.44627 |
| Cl | (0,2) 3p²²¹ | 0.5 | -1.15124 | 1.77235 | -1.82143 | -0.36457 | 1.15347 | -1.52700 | -1.55609 | 1.42704 |
|  | (+1,1) 1s¹3p²²¹ | 0.5 | -1.07375 | 1.65121 | -1.75236 | -0.51233 | 1.51332 | -1.87449 | -1.67046 | 1.38855 |
|  | (+1,3) 1s¹3p²²¹ | 0.5 | -1.08753 | 1.70588 | -1.80738 | -0.51680 | 1.50957 | -1.88174 | -1.65333 | 1.40165 |
|  | (+1,1) 2s¹3p²²¹ | 0.5 | -1.20761 | 2.11597 | -2.04420 | -0.47166 | 1.36220 | -1.79357 | -1.33171 | 1.31198 |
|  | (+1,3) 2s¹3p²²¹ | 0.5 | -1.21448 | 2.14316 | -2.07104 | -0.47183 | 1.35976 | -1.78564 | -1.32531 | 1.30727 |
|  | (+1,1) 2p¹²²3p²²¹ | 0.5 | -1.33144 | 2.37843 | -2.34359 | -0.32243 | 1.01714 | -1.51925 | -1.67863 | 1.45280 |
|  | (+1,3) 2p¹²²3p²²¹ | 0.5 | -1.33017 | 2.37527 | -2.33309 | -0.32505 | 1.01376 | -1.50851 | -1.65037 | 1.43865 |
|  | (+1,1) 2p²²¹3p²²¹ | 0.5 | -1.33358 | 2.38074 | -2.34744 | -0.32570 | 1.02097 | -1.53260 | -1.67788 | 1.46861 |
|  | (+1,3) 2p²²¹3p²²¹ | 0.5 | -1.32257 | 2.34779 | -2.29742 | -0.34360 | 1.01604 | -1.51523 | -1.55188 | 1.42895 |
|  | (+1,1) 3s¹3p²²¹ | 0.5 | -1.21739 | 1.97833 | -2.03017 | -0.41346 | 1.28864 | -1.76659 | -1.60204 | 1.53793 |
|  | (+1,3) 3s¹3p²²¹ | 0.5 | -1.22798 | 2.04219 | -2.08005 | -0.40837 | 1.25348 | -1.69687 | -1.55122 | 1.48267 |
|  | (+1,1) 3p¹²¹ | 0.5 | -1.27735 | 2.23172 | -2.24507 | -0.38098 | 1.19620 | -1.61644 | -1.59862 | 1.44937 |
|  | (+1,3) 3p¹²¹ | 0.5 | -1.27610 | 2.22991 | -2.23077 | -0.38042 | 1.19124 | -1.59938 | -1.57730 | 1.42414 |
|  | (+1,1) 3p²²⁰ | 0.5 | -1.26322 | 2.17019 | -2.19112 | -0.37814 | 1.19883 | -1.61959 | -1.62045 | 1.45729 |
| Ar | (0,1) 3p²²² | 0.5 | -1.04138 | 1.31159 | -1.41416 | -0.37265 | 1.26868 | -1.69946 | -1.60444 | 1.46043 |
|  | (+1,2) 1s¹3p²²² | 0.5 | -0.96429 | 1.20999 | -1.36102 | -0.51064 | 1.57085 | -1.98404 | -1.65989 | 1.40694 |
|  | (+1,2) 2s¹3p²²² | 0.5 | -1.06613 | 1.55071 | -1.56276 | -0.47808 | 1.47833 | -1.96159 | -1.38630 | 1.35097 |
|  | (+1,2) 2p¹²²3p²²² | 0.5 | -1.23686 | 1.99657 | -1.99854 | -0.33771 | 1.10367 | -1.64336 | -1.65716 | 1.46368 |
|  | (+1,2) 3s¹3p²²² | 0.5 | -1.13011 | 1.61874 | -1.68111 | -0.42248 | 1.37967 | -1.89393 | -1.56302 | 1.50974 |
|  | (+1,2) 3p²²¹ | 0.5 | -1.16574 | 1.76392 | -1.80594 | -0.39426 | 1.30839 | -1.77377 | -1.57661 | 1.44595 |

### **Table S.4.** Optimized parameters for the coefficients of the explicit Boys–Handy correlation function for the elements from He to F using DZ basis set. Values in parenthesis are the charge and multiplicity.

| Atom | System | $c_{1A}$ | $c_{2A}$ | $c_{3A}$ | $c_{4A}$ | $c_{5A}$ | $c_{6A}$ | $c_{7A}$ | $c_{8A}$ | $c_{9A}$ |
| --- | --- | --- | --- | --- | --- | --- | --- | --- | --- | --- |
| He | (0,1) 1s² | 0.5 | 0.00658 | 0.14107 | -0.21120 | 0.22143 | 0.03704 | -1.04136 | -2.58433 | 0.91270 |
| Li | (0,2) 2s¹ | 0.5 | 0.02881 | 0.54878 | -1.04169 | -0.06357 | 0.19583 | -0.55257 | -1.04796 | 0.63659 |
|  | (+1,1) 1s¹2s¹ | 0.5 | -0.05737 | 0.56930 | -0.88443 | -0.70434 | 1.17697 | -4.39418 | -0.33841 | 0.39067 |
|  | (+1,3) 1s¹2s¹ | 0.5 | -4.84910 | 11.33811 | -7.72655 | -1.19303 | 3.59341 | -6.73241 | -0.81343 | 0.64161 |
|  | (+1,1) 1s² | 0.5 | -0.05142 | 0.14500 | -0.42061 | 0.05375 | -0.31603 | -0.49654 | -3.08972 | 0.95092 |
| Be | (0,1) 2s² | 0.5 | 0.39917 | -1.69405 | 0.14430 | -0.34207 | 1.32932 | -2.16977 | -2.23599 | 2.17995 |
|  | (+1,2) 1s¹2s² | 0.5 | -0.74799 | 0.77779 | -1.36522 | -0.68165 | 2.44776 | -4.52837 | -2.76971 | 2.40668 |
|  | (+1,2) 2s¹ | 0.5 | -0.08255 | 0.51560 | -1.01948 | -0.45107 | 1.44311 | -2.07715 | -0.74523 | 0.53176 |
| B | (0,2) 2p¹⁰⁰ | 0.5 | 0.11000 | -1.23436 | 0.06461 | -0.24732 | 1.02342 | -1.84443 | -2.06646 | 1.98115 |
|  | (+1,1) 1s2p¹⁰⁰ | 0.5 | -1.33170 | 2.62353 | -2.60604 | -0.47177 | 1.50930 | -2.97576 | -2.26442 | 1.95273 |
|  | (+1,3) 1s2p¹⁰⁰ | 0.5 | -1.60982 | 3.40071 | -3.10745 | -0.44267 | 1.15675 | -2.62442 | -2.04800 | 1.84776 |
|  | (+1,1) 2s¹2p¹⁰⁰ | 0.5 | 0.00641 | -0.84387 | -0.35244 | -0.44903 | 1.79050 | -3.03733 | -2.12878 | 2.12500 |
|  | (+1,3) 2s¹2p¹⁰⁰ | 0.5 | -0.07259 | -0.30732 | -0.39806 | -0.39802 | 1.43391 | -2.28252 | -1.15582 | 1.09673 |
|  | (+1,1) 2s² | 0.5 | 0.06059 | -1.06623 | -0.20071 | -0.43945 | 1.80707 | -3.04412 | -2.24272 | 2.19988 |
| C | (0,3) 2p¹¹⁰ | 0.5 | -0.23530 | -0.41297 | -0.37287 | -0.12732 | 0.64641 | -1.42844 | -1.96880 | 1.75607 |
|  | (+1,2) 1s¹2p¹¹⁰ | 0.5 | -1.33341 | 2.78606 | -2.69869 | -0.34781 | 0.98797 | -2.13928 | -2.00449 | 1.65135 |
|  | (+1,4) 1s¹2p¹¹⁰ | 0.5 | -1.71660 | 3.87757 | -3.46319 | -0.30970 | 0.59935 | -1.71374 | -1.81290 | 1.54380 |
|  | (+1,2) 2s¹2p¹¹⁰ | 0.5 | -0.25753 | -0.45338 | -0.43609 | -0.31809 | 1.37045 | -2.62867 | -2.19605 | 2.12768 |
|  | (+1,4) 2s¹2p¹¹⁰ | 0.5 | -0.30994 | -0.05755 | -0.48258 | -0.25542 | 1.04488 | -1.93230 | -1.39252 | 1.22747 |
|  | (+1,2) 2p¹⁰⁰ | 0.5 | -0.31857 | -0.14158 | -0.69302 | -0.30010 | 1.21380 | -2.35548 | -1.97624 | 1.94036 |
| N | (0,4) 2p¹¹¹ | 0.5 | -0.50873 | 0.29458 | -0.79478 | -0.03241 | 0.33809 | -1.05282 | -1.93468 | 1.55532 |
|  | (+1,3) 1s¹2p¹¹¹ | 0.5 | -1.20581 | 2.51485 | -2.45685 | -0.26166 | 0.67524 | -1.61618 | -1.87171 | 1.40234 |
|  | (+1,5) 1s¹2p¹¹¹ | 0.5 | -1.57421 | 3.59165 | -3.25207 | -0.23438 | 0.34660 | -1.21500 | -1.71734 | 1.30494 |
|  | (+1,3) 2s¹2p¹¹¹ | 0.5 | -0.53473 | 0.18818 | -0.74898 | -0.20648 | 0.98230 | -2.19429 | -2.18752 | 1.98472 |
|  | (+1,5) 2s¹2p¹¹¹ | 0.5 | -0.60505 | 0.58870 | -0.89012 | -0.13247 | 0.66797 | -1.51103 | -1.53559 | 1.19438 |
|  | (+1,3) 2p¹¹⁰ | 0.5 | -0.62276 | 0.65593 | -1.17828 | -0.16782 | 0.72088 | -1.75388 | -1.89763 | 1.72139 |
| O | (0,3) 2p²¹¹ | 0.5 | -0.67523 | 0.70661 | -1.13806 | 0.01396 | 0.25500 | -0.96846 | -2.14843 | 1.59257 |
|  | (+1,2) 1s¹2p²¹¹ | 0.5 | -1.11683 | 2.28885 | -2.40263 | -0.23135 | 0.60806 | -1.45824 | -2.03517 | 1.45228 |
|  | (+1,4) 1s¹2p²¹¹ | 0.5 | -1.24315 | 2.67826 | -2.69882 | -0.22482 | 0.48885 | -1.28420 | -1.96544 | 1.39172 |
|  | (+1,2) 2s¹2p²¹¹ | 0.5 | -0.80294 | 1.02655 | -1.37881 | -0.11548 | 0.66688 | -1.77471 | -2.21633 | 1.81987 |
|  | (+1,4) 2s¹2p²¹¹ | 0.5 | -0.84096 | 1.24820 | -1.57199 | -0.07940 | 0.50365 | -1.45305 | -2.00405 | 1.55710 |
|  | (+1,2) 2p¹¹¹ | 0.5 | -0.86054 | 1.31204 | -1.69375 | -0.09653 | 0.50750 | -1.48973 | -2.02348 | 1.68212 |
|  | (+1,4) 2p¹¹¹ | 0.5 | -0.87690 | 1.37657 | -1.66670 | -0.09621 | 0.48288 | -1.41929 | -1.87370 | 1.53121 |
|  | (+1,2) 2p²¹⁰ | 0.5 | -0.83284 | 1.19990 | -1.62995 | -0.09324 | 0.52774 | -1.53411 | -2.12827 | 1.76461 |
| F | (0,2) 2p²²¹ | 0.5 | -0.74736 | 0.85727 | -1.26110 | 0.03532 | 0.21276 | -0.90636 | -2.29904 | 1.58904 |
|  | (+1,1) 1s2p²²¹ | 0.5 | -0.99755 | 1.90968 | -2.14359 | -0.21333 | 0.59324 | -1.36805 | -2.15557 | 1.43670 |
|  | (+1,3) 1s2p²²¹ | 0.5 | -1.02842 | 2.00622 | -2.21429 | -0.21324 | 0.55870 | -1.30648 | -2.12777 | 1.40878 |
|  | (+1,1) 2s2p²²¹ | 0.5 | -0.91887 | 1.39530 | -1.66113 | -0.06550 | 0.47510 | -1.48914 | -2.26828 | 1.69927 |
|  | (+1,3) 2s2p²²¹ | 0.5 | -0.93543 | 1.49347 | -1.76571 | -0.05061 | 0.40690 | -1.35416 | -2.19937 | 1.60989 |
|  | (+1,1) 2p²¹¹ | 0.5 | -0.94692 | 1.51181 | -1.85589 | -0.05317 | 0.39350 | -1.32725 | -2.19268 | 1.66834 |
|  | (+1,3) 2p²¹¹ | 0.5 | -0.95021 | 1.52753 | -1.83908 | -0.05186 | 0.38332 | -1.29804 | -2.13970 | 1.60894 |
|  | (+1,1) 2p²²⁰ | 0.5 | -0.93198 | 1.44678 | -1.83399 | -0.05105 | 0.41040 | -1.37009 | -2.28633 | 1.74855 |

### **Table S.5.** Optimized parameters for the coefficients of the explicit Boys–Handy correlation function for the elements from Ne to Si using DZ basis set. Values in parenthesis are the charge and multiplicity.

| Atom | System | $c_{1A}$ | $c_{2A}$ | $c_{3A}$ | $c_{4A}$ | $c_{5A}$ | $c_{6A}$ | $c_{7A}$ | $c_{8A}$ | $c_{9A}$ |
| --- | --- | --- | --- | --- | --- | --- | --- | --- | --- | --- |
| Ne | (0,1) 2p²²² | 0.5 | -0.76493 | 0.84854 | -1.24063 | 0.04367 | 0.19537 | -0.85623 | -2.41573 | 1.55786 |
|  | (+1,2) 1s2p²²² | 0.5 | -0.88168 | 1.49393 | -1.79549 | -0.20151 | 0.59645 | -1.30275 | -2.25370 | 1.38573 |
|  | (+1,2) 2s2p²²² | 0.5 | -0.95984 | 1.52757 | -1.75946 | -0.03846 | 0.35701 | -1.27883 | -2.32374 | 1.59086 |
|  | (+1,2) 2p²²¹ | 0.5 | -0.96769 | 1.51963 | -1.84608 | -0.02929 | 0.32718 | -1.20233 | -2.31898 | 1.61999 |
| Na | (0,2) 3s¹ | 0.5 | -0.93054 | 1.39896 | -1.72583 | 0.03109 | 0.11308 | -0.69123 | -2.26700 | 1.54510 |
|  | (+1,1) 1s¹3s¹ | 0.5 | -0.96624 | 1.77613 | -2.03607 | -0.19766 | 0.49981 | -1.13330 | -2.07651 | 1.35899 |
|  | (+1,3) 1s¹3s¹ | 0.5 | -0.98944 | 1.85557 | -2.10125 | -0.20182 | 0.50799 | -1.14159 | -2.06369 | 1.35426 |
|  | (+1,1) 2s¹3s¹ | 0.5 | -1.20797 | 2.38141 | -2.52041 | -0.05892 | 0.25670 | -1.08909 | -2.10671 | 1.57424 |
|  | (+1,3) 2s¹3s¹ | 0.5 | -1.24861 | 2.51531 | -2.62495 | -0.06719 | 0.27786 | -1.11946 | -2.07965 | 1.56825 |
|  | (+1,1) 2p²²¹3s¹ | 0.5 | -1.21020 | 2.34630 | -2.55734 | -0.05391 | 0.24449 | -1.03653 | -2.08494 | 1.58163 |
|  | (+1,3) 2p²²¹3s¹ | 0.5 | -1.22470 | 2.39616 | -2.59611 | -0.05628 | 0.25007 | -1.04266 | -2.07431 | 1.57598 |
|  | (+1,1) 2p²²² | 0.5 | -0.84771 | 1.06602 | -1.43161 | 0.01809 | 0.12750 | -0.78476 | -2.48133 | 1.57201 |
| Mg | (0,1) 3s² | 0.5 | -0.99176 | 1.52188 | -1.84930 | -0.02270 | 0.22277 | -0.81674 | -2.21295 | 1.64220 |
|  | (+1,2) 1s¹3s² | 0.5 | -0.91394 | 1.48554 | -1.79335 | -0.20980 | 0.54433 | -1.18948 | -2.09593 | 1.46958 |
|  | (+1,2) 2s¹3s² | 0.5 | -1.18125 | 2.19591 | -2.34456 | -0.08845 | 0.34017 | -1.17692 | -2.11018 | 1.64451 |
|  | (+1,2) 2p⁵3s² | 0.5 | -1.16757 | 2.09029 | -2.31174 | -0.08489 | 0.33241 | -1.13530 | -2.08940 | 1.65694 |
|  | (+1,2) 2s3s | 0.5 | -1.13789 | 2.07680 | -2.33370 | -0.02624 | 0.13218 | -0.72028 | -2.20807 | 1.55290 |
| Al | (0,2) 3p¹⁰⁰ | 0.5 | -1.04746 | 1.66444 | -1.96338 | -0.05394 | 0.25284 | -0.81229 | -2.11640 | 1.64642 |
|  | (+1,1) 1s¹3p¹⁰⁰ | 0.5 | -0.97172 | 1.64446 | -1.91968 | -0.20921 | 0.45991 | -1.03917 | -1.98045 | 1.46074 |
|  | (+1,3) 1s¹3p¹⁰⁰ | 0.5 | -0.98447 | 1.69031 | -1.95874 | -0.21074 | 0.45896 | -1.03775 | -1.97454 | 1.45969 |
|  | (+1,1) 2s¹3p¹⁰⁰ | 0.5 | -1.22939 | 2.33872 | -2.44361 | -0.09709 | 0.28079 | -1.03369 | -1.99128 | 1.59570 |
|  | (+1,3) 2s¹3p¹⁰⁰ | 0.5 | -1.23116 | 2.34922 | -2.44647 | -0.09754 | 0.27557 | -1.02364 | -1.97448 | 1.58310 |
|  | (+1,1) 2p¹²²3p¹⁰⁰ | 0.5 | -1.20953 | 2.18815 | -2.37488 | -0.08570 | 0.28783 | -1.02363 | -2.04600 | 1.63806 |
|  | (+1,3) 2p¹²²3p¹⁰⁰ | 0.5 | -1.22410 | 2.24386 | -2.41281 | -0.09971 | 0.29555 | -1.03098 | -1.97202 | 1.62003 |
|  | (+1,1) 2p²¹²3p¹⁰⁰ | 0.5 | -1.21668 | 2.21425 | -2.38532 | -0.09714 | 0.29366 | -1.02802 | -1.98340 | 1.62199 |
|  | (+1,3) 2p²¹²3p¹⁰⁰ | 0.5 | -1.21549 | 2.20869 | -2.37853 | -0.09759 | 0.29538 | -1.03078 | -1.98266 | 1.62161 |
|  | (+1,1) 3s⁰3p¹⁰⁰ | 0.5 | -1.18695 | 2.15616 | -2.39958 | -0.06404 | 0.23608 | -0.84537 | -2.16926 | 1.64019 |
|  | (+1,3) 3s⁰3p¹⁰⁰ | 0.5 | -1.22449 | 2.36460 | -2.55183 | -0.05389 | 0.14694 | -0.66283 | -2.02161 | 1.48273 |
|  | (+1,1) 3s² | 0.5 | -1.15049 | 2.02516 | -2.26270 | -0.06435 | 0.23890 | -0.84096 | -2.15853 | 1.61385 |
| Si | (0,3) 3p¹¹⁰ | 0.5 | -1.03880 | 1.58041 | -1.84517 | -0.09332 | 0.34183 | -0.87129 | -1.98903 | 1.59833 |
|  | (+1,2) 1s¹3p¹¹⁰ | 0.5 | -0.96367 | 1.54694 | -1.80978 | -0.23087 | 0.51361 | -1.05521 | -1.89739 | 1.45091 |
|  | (+1,4) 1s¹3p¹¹⁰ | 0.5 | -0.96544 | 1.56296 | -1.81458 | -0.23333 | 0.51427 | -1.04867 | -1.87135 | 1.42835 |
|  | (+1,2) 2s¹3p¹¹⁰ | 0.5 | -1.19777 | 2.19289 | -2.27934 | -0.12309 | 0.32565 | -1.02269 | -1.87641 | 1.53881 |
|  | (+1,4) 2s¹3p¹¹⁰ | 0.5 | -1.19809 | 2.20555 | -2.27835 | -0.12526 | 0.31890 | -1.00236 | -1.83313 | 1.50546 |
|  | (+1,2) 2p¹²²3p¹¹⁰ | 0.5 | -1.19398 | 2.08243 | -2.23849 | -0.10988 | 0.33597 | -1.02297 | -1.94764 | 1.58882 |
|  | (+1,4) 2p¹²²3p¹¹⁰ | 0.5 | -1.19397 | 2.08398 | -2.22302 | -0.12621 | 0.34877 | -1.03371 | -1.85983 | 1.56297 |
|  | (+1,2) 2p²²¹3p¹¹⁰ | 0.5 | -1.19600 | 2.08707 | -2.22828 | -0.12237 | 0.34304 | -1.02973 | -1.88096 | 1.57323 |
|  | (+1,4) 2p²²¹3p¹¹⁰ | 0.5 | -1.19588 | 2.09096 | -2.22363 | -0.12356 | 0.34042 | -1.02176 | -1.86054 | 1.55746 |
|  | (+1,2) 3s¹3p¹¹⁰ | 0.5 | -1.15605 | 1.95667 | -2.20267 | -0.10881 | 0.38430 | -1.01667 | -2.11863 | 1.69065 |
|  | (+1,4) 3s¹3p¹¹⁰ | 0.5 | -1.17735 | 2.15367 | -2.30283 | -0.09702 | 0.26236 | -0.73636 | -1.84604 | 1.40109 |
|  | (+1,2) 3p¹⁰⁰ | 0.5 | -1.14814 | 1.96637 | -2.18749 | -0.10394 | 0.33815 | -0.91684 | -2.03631 | 1.59867 |

### **Table S.6.** Optimized parameters for the coefficients of the explicit Boys–Handy correlation function for the elements from P to Ar using DZ basis set. Values in parenthesis are the charge and multiplicity.

| Atom | System | $c_{1A}$ | $c_{2A}$ | $c_{3A}$ | $c_{4A}$ | $c_{5A}$ | $c_{6A}$ | $c_{7A}$ | $c_{8A}$ | $c_{9A}$ |
| --- | --- | --- | --- | --- | --- | --- | --- | --- | --- | --- |
| P | (0,4) 3p¹¹¹ | 0.5 | -1.02435 | 1.47099 | -1.68671 | -0.12755 | 0.42227 | -0.92299 | -1.84786 | 1.52479 |
|  | (+1,3) 1s¹3p¹¹¹ | 0.5 | -0.96045 | 1.47200 | -1.69910 | -0.24908 | 0.56009 | -1.06314 | -1.79374 | 1.40471 |
|  | (+1,5) 1s¹3p¹¹¹ | 0.5 | -0.95564 | 1.46198 | -1.67959 | -0.25357 | 0.56924 | -1.06754 | -1.76754 | 1.38340 |
|  | (+1,3) 2s¹3p¹¹¹ | 0.5 | -1.17108 | 2.06252 | -2.11507 | -0.14644 | 0.36964 | -1.01064 | -1.74734 | 1.45656 |
|  | (+1,5) 2s¹3p¹¹¹ | 0.5 | -1.16669 | 2.05486 | -2.09649 | -0.14964 | 0.36772 | -0.99833 | -1.70472 | 1.42637 |
|  | (+1,3) 2p¹²²3p¹¹¹ | 0.5 | -1.17538 | 1.96550 | -2.07923 | -0.13349 | 0.38760 | -1.03054 | -1.83462 | 1.52005 |
|  | (+1,5) 2p¹²²3p¹¹¹ | 0.5 | -1.17929 | 1.98048 | -2.06960 | -0.14686 | 0.38772 | -1.02111 | -1.73595 | 1.48089 |
|  | (+1,3) 3s¹3p¹¹¹ | 0.5 | -1.13084 | 1.77790 | -2.00659 | -0.14649 | 0.51272 | -1.15893 | -2.05400 | 1.70325 |
|  | (+1,5) 3s¹3p¹¹¹ | 0.5 | -1.13741 | 1.95153 | -2.04418 | -0.13338 | 0.36956 | -0.81033 | -1.67974 | 1.30476 |
|  | (+1,3) 3p¹¹⁰ | 0.5 | -1.12974 | 1.84403 | -2.02875 | -0.13743 | 0.42901 | -0.98120 | -1.90348 | 1.54387 |
| S | (0,3) 3p²¹¹ | 0.5 | -1.04835 | 1.46992 | -1.67114 | -0.14508 | 0.48242 | -0.99509 | -1.83894 | 1.55387 |
|  | (+1,2) 1s¹3p²¹¹ | 0.5 | -0.99012 | 1.48514 | -1.70618 | -0.25762 | 0.60523 | -1.11041 | -1.80241 | 1.44998 |
|  | (+1,4) 1s¹3p²¹¹ | 0.5 | -0.98770 | 1.47891 | -1.69589 | -0.25988 | 0.61047 | -1.11417 | -1.79083 | 1.44082 |
|  | (+1,2) 2s¹3p²¹¹ | 0.5 | -1.17683 | 2.02361 | -2.07629 | -0.16044 | 0.40459 | -1.03178 | -1.72318 | 1.47058 |
|  | (+1,4) 2s¹3p²¹¹ | 0.5 | -1.17330 | 2.01563 | -2.06799 | -0.16128 | 0.40264 | -1.02584 | -1.70895 | 1.46067 |
|  | (+1,2) 2p¹²²3p²¹¹ | 0.5 | -1.19278 | 1.95846 | -2.06361 | -0.14664 | 0.42298 | -1.05671 | -1.81973 | 1.53921 |
|  | (+1,4) 2p¹²²3p²¹¹ | 0.5 | -1.19364 | 1.96343 | -2.06038 | -0.14559 | 0.41914 | -1.04833 | -1.80909 | 1.52493 |
|  | (+1,2) 2p²¹²3p²¹¹ | 0.5 | -1.19681 | 1.97206 | -2.07674 | -0.14540 | 0.41982 | -1.05330 | -1.82502 | 1.54226 |
|  | (+1,4) 2p²¹²3p²¹¹ | 0.5 | -1.19487 | 1.95928 | -2.05163 | -0.15853 | 0.42702 | -1.05995 | -1.75206 | 1.52600 |
|  | (+1,2) 3s¹3p⁴ | 0.5 | -1.14876 | 1.77278 | -1.96259 | -0.16611 | 0.56822 | -1.20870 | -1.98324 | 1.68556 |
|  | (+1,2) 3s¹3p⁴ | 0.5 | -1.14811 | 1.84742 | -1.98130 | -0.15990 | 0.49970 | -1.03889 | -1.80105 | 1.49288 |
|  | (+1,2) 3p¹¹¹ | 0.5 | -1.15172 | 1.84881 | -2.00348 | -0.15792 | 0.49195 | -1.04608 | -1.84610 | 1.54251 |
|  | (+1,4) 3p¹¹¹ | 0.5 | -1.15585 | 1.87598 | -2.00349 | -0.15801 | 0.47934 | -1.01202 | -1.79265 | 1.49039 |
|  | (+1,2) 3p²¹⁰ | 0.5 | -1.13994 | 1.79613 | -1.97982 | -0.15741 | 0.49939 | -1.06799 | -1.89080 | 1.58704 |
| Cl | (0,2) 3p²²¹ | 0.5 | -1.07408 | 1.48780 | -1.65380 | -0.15840 | 0.53317 | -1.04756 | -1.80937 | 1.54401 |
|  | (+1,1) 1s¹3p²²¹ | 0.5 | -1.01719 | 1.50763 | -1.70253 | -0.26610 | 0.64314 | -1.13980 | -1.77181 | 1.45028 |
|  | (+1,3) 1s¹3p²²¹ | 0.5 | -1.01596 | 1.50460 | -1.69878 | -0.26663 | 0.64633 | -1.14275 | -1.77117 | 1.44768 |
|  | (+1,1) 2s¹3p²²¹ | 0.5 | -1.18705 | 2.00488 | -2.05096 | -0.17111 | 0.43914 | -1.04941 | -1.70144 | 1.46821 |
|  | (+1,3) 2s¹3p²²¹ | 0.5 | -1.18802 | 2.01194 | -2.05563 | -0.17194 | 0.43562 | -1.04325 | -1.68782 | 1.46073 |
|  | (+1,1) 2p¹²²3p²²¹ | 0.5 | -1.21159 | 1.96551 | -2.04753 | -0.15729 | 0.45562 | -1.07546 | -1.79208 | 1.53122 |
|  | (+1,3) 2p¹²²3p²²¹ | 0.5 | -1.21500 | 1.97877 | -2.05383 | -0.15761 | 0.45426 | -1.07218 | -1.78199 | 1.52290 |
|  | (+1,1) 2p²²¹3p²²¹ | 0.5 | -1.21770 | 1.98740 | -2.06611 | -0.15633 | 0.45334 | -1.07264 | -1.79591 | 1.53253 |
|  | (+1,3) 2p²²¹3p²²¹ | 0.5 | -1.21847 | 1.98172 | -2.05045 | -0.16804 | 0.45778 | -1.07807 | -1.73039 | 1.52258 |
|  | (+1,1) 3s¹3p²²¹ | 0.5 | -1.16930 | 1.78789 | -1.93193 | -0.18107 | 0.61175 | -1.23961 | -1.90575 | 1.64454 |
|  | (+1,3) 3s¹3p²²¹ | 0.5 | -1.16695 | 1.80777 | -1.93737 | -0.17923 | 0.58692 | -1.17871 | -1.84147 | 1.57960 |
|  | (+1,1) 3p¹²¹ | 0.5 | -1.16563 | 1.82548 | -1.96071 | -0.17358 | 0.54773 | -1.10604 | -1.81113 | 1.54758 |
|  | (+1,3) 3p¹²¹ | 0.5 | -1.16814 | 1.83861 | -1.96067 | -0.17305 | 0.54403 | -1.09527 | -1.79327 | 1.52592 |
|  | (+1,1) 3p²²⁰ | 0.5 | -1.15978 | 1.79503 | -1.94907 | -0.17345 | 0.55590 | -1.12573 | -1.84596 | 1.58083 |
| Ar | (0,1) 3p²²² | 0.5 | -1.09793 | 1.51238 | -1.64252 | -0.16835 | 0.57136 | -1.08227 | -1.77247 | 1.51974 |
|  | (+1,2) 1s¹3p²²² | 0.5 | -1.03599 | 1.51171 | -1.67979 | -0.27181 | 0.67892 | -1.16790 | -1.74376 | 1.43728 |
|  | (+1,2) 2s¹3p²²² | 0.5 | -1.18973 | 1.96565 | -1.99671 | -0.18150 | 0.46836 | -1.06111 | -1.65870 | 1.44626 |
|  | (+1,2) 2p¹²²3p²²² | 0.5 | -1.23223 | 1.98825 | -2.04195 | -0.16546 | 0.48310 | -1.08667 | -1.75750 | 1.50773 |
|  | (+1,2) 3s¹3p²²² | 0.5 | -1.18107 | 1.77505 | -1.87346 | -0.19328 | 0.64822 | -1.26714 | -1.83291 | 1.60011 |
|  | (+1,2) 3p²²¹ | 0.5 | -1.18071 | 1.81700 | -1.91817 | -0.18444 | 0.59266 | -1.14786 | -1.76765 | 1.52322 |

### **Table S.7.** Optimized parameters for the coefficients of the explicit Boys–Handy correlation function for the elements from He to F using NHF basis set. Values in parenthesis are the charge and multiplicity.

| Atom | System | $c_{1A}$ | $c_{2A}$ | $c_{3A}$ | $c_{4A}$ | $c_{5A}$ | $c_{6A}$ | $c_{7A}$ | $c_{8A}$ | $c_{9A}$ |
| --- | --- | --- | --- | --- | --- | --- | --- | --- | --- | --- |
| He | (0,1) 1s² | 0.5 | -0.01439 | 0.20565 | -0.26799 | 0.24805 | -0.06584 | -0.94484 | -2.58597 | 0.91862 |
| Li | (0,2) 2s¹ | 0.5 | 0.02998 | 0.54971 | -1.04176 | -0.06308 | 0.19061 | -0.54414 | -1.04045 | 0.63060 |
|  | (+1,1) 1s¹2s¹ | 0.5 | -0.03059 | 0.48691 | -0.81372 | -0.70182 | 1.14446 | -4.37298 | -0.30979 | 0.37673 |
|  | (+1,3) 1s¹2s¹ | 0.5 | -4.78125 | 11.17638 | -7.61967 | -1.18802 | 3.56124 | -6.69978 | -0.80372 | 0.63671 |
|  | (+1,1) 1s² | 0.5 | -0.05335 | 0.15147 | -0.42684 | 0.05453 | -0.32253 | -0.48531 | -3.09251 | 0.95163 |
| Be | (0,1) 2s² | 0.5 | 0.40178 | -1.69850 | 0.14586 | -0.34119 | 1.32549 | -2.16314 | -2.23679 | 2.17915 |
|  | (+1,2) 1s¹2s² | 0.5 | -0.76721 | 0.82985 | -1.40617 | -0.68163 | 2.45114 | -4.52959 | -2.77857 | 2.41259 |
|  | (+1,2) 2s¹ | 0.5 | -0.08479 | 0.52135 | -1.02339 | -0.45214 | 1.44071 | -2.07206 | -0.74237 | 0.53204 |
| B | (0,2) 2p¹⁰⁰ | 0.5 | 0.11904 | -1.26131 | 0.08962 | -0.24047 | 1.00275 | -1.82439 | -2.06247 | 1.97232 |
|  | (+1,1) 1s2p¹⁰⁰ | 0.5 | -1.24035 | 2.40789 | -2.43891 | -0.45299 | 1.45321 | -2.92553 | -2.24182 | 1.91739 |
|  | (+1,3) 1s2p¹⁰⁰ | 0.5 | -1.56821 | 3.27145 | -3.00133 | -0.42118 | 1.12311 | -2.60883 | -2.07199 | 1.84381 |
|  | (+1,1) 2s¹2p¹⁰⁰ | 0.5 | 0.03110 | -0.93769 | -0.27456 | -0.44021 | 1.77759 | -3.03491 | -2.14469 | 2.12848 |
|  | (+1,3) 2s¹2p¹⁰⁰ | 0.5 | -0.04181 | -0.41745 | -0.28937 | -0.38590 | 1.41078 | -2.26716 | -1.15257 | 1.07626 |
|  | (+1,1) 2s² | 0.5 | 0.05900 | -1.06088 | -0.20686 | -0.44237 | 1.81552 | -3.05448 | -2.24378 | 2.20451 |
| C | (0,3) 2p¹¹⁰ | 0.5 | -0.24116 | -0.39797 | -0.38418 | -0.11409 | 0.60625 | -1.39350 | -1.97895 | 1.75667 |
|  | (+1,2) 1s¹2p¹¹⁰ | 0.5 | -1.20141 | 2.46048 | -2.44567 | -0.30524 | 0.86487 | -2.03237 | -1.99810 | 1.61261 |
|  | (+1,4) 1s¹2p¹¹⁰ | 0.5 | -1.65095 | 3.68359 | -3.30356 | -0.26101 | 0.48305 | -1.62652 | -1.85002 | 1.53620 |
|  | (+1,2) 2s¹2p¹¹⁰ | 0.5 | -0.24521 | -0.50749 | -0.39591 | -0.29921 | 1.32534 | -2.59885 | -2.22876 | 2.14018 |
|  | (+1,4) 2s¹2p¹¹⁰ | 0.5 | -0.28865 | -0.13453 | -0.39869 | -0.23253 | 0.98093 | -1.87462 | -1.39391 | 1.20251 |
|  | (+1,2) 2p¹⁰⁰ | 0.5 | -0.31210 | -0.16830 | -0.66729 | -0.28982 | 1.18359 | -2.33103 | -1.98484 | 1.94004 |
| N | (0,4) 2p¹¹¹ | 0.5 | -0.53896 | 0.38980 | -0.87206 | -0.01229 | 0.26445 | -0.98202 | -1.94180 | 1.55435 |
|  | (+1,3) 1s¹2p¹¹¹ | 0.5 | -1.11375 | 2.30184 | -2.28619 | -0.21137 | 0.50334 | -1.45349 | -1.85708 | 1.36143 |
|  | (+1,5) 1s¹2p¹¹¹ | 0.5 | -1.55031 | 3.52443 | -3.19143 | -0.17864 | 0.18061 | -1.06874 | -1.74176 | 1.29301 |
|  | (+1,3) 2s¹2p¹¹¹ | 0.5 | -0.55153 | 0.23283 | -0.79332 | -0.18162 | 0.89935 | -2.12277 | -2.21455 | 1.99946 |
|  | (+1,5) 2s¹2p¹¹¹ | 0.5 | -0.60082 | 0.56853 | -0.86174 | -0.10462 | 0.57203 | -1.42139 | -1.54683 | 1.18489 |
|  | (+1,3) 2p¹¹⁰ | 0.5 | -0.63163 | 0.68393 | -1.19941 | -0.14982 | 0.65405 | -1.69064 | -1.90233 | 1.71840 |
| O | (0,3) 2p²¹¹ | 0.5 | -0.74290 | 0.92920 | -1.32996 | 0.03842 | 0.15434 | -0.86870 | -2.15676 | 1.59975 |
|  | (+1,2) 1s¹2p²¹¹ | 0.5 | -1.08406 | 2.24283 | -2.36929 | -0.17222 | 0.39400 | -1.25235 | -2.02581 | 1.42413 |
|  | (+1,4) 1s¹2p²¹¹ | 0.5 | -1.24674 | 2.71147 | -2.72574 | -0.16440 | 0.28014 | -1.09187 | -1.97396 | 1.38115 |
|  | (+1,2) 2s¹2p²¹¹ | 0.5 | -0.84301 | 1.15021 | -1.49917 | -0.08350 | 0.55046 | -1.67047 | -2.25219 | 1.84672 |
|  | (+1,4) 2s¹2p²¹¹ | 0.5 | -0.87089 | 1.34502 | -1.66017 | -0.04324 | 0.37149 | -1.32732 | -2.03255 | 1.56731 |
|  | (+1,2) 2p¹¹¹ | 0.5 | -0.88429 | 1.39317 | -1.76371 | -0.06978 | 0.40565 | -1.38744 | -2.03793 | 1.68178 |
|  | (+1,4) 2p¹¹¹ | 0.5 | -0.90006 | 1.45613 | -1.73304 | -0.07132 | 0.38682 | -1.32332 | -1.88196 | 1.52783 |
|  | (+1,2) 2p²¹⁰ | 0.5 | -0.85940 | 1.29041 | -1.71125 | -0.06746 | 0.42860 | -1.43890 | -2.14565 | 1.77119 |
| F | (0,2) 2p²²¹ | 0.5 | -0.85838 | 1.23239 | -1.58903 | 0.06281 | 0.08009 | -0.76712 | -2.29248 | 1.59669 |
|  | (+1,1) 1s2p²²¹ | 0.5 | -1.03402 | 2.07635 | -2.29303 | -0.14933 | 0.33396 | -1.10802 | -2.13843 | 1.41982 |
|  | (+1,3) 1s2p²²¹ | 0.5 | -1.08434 | 2.22352 | -2.40570 | -0.14878 | 0.30316 | -1.05353 | -2.11741 | 1.39807 |
|  | (+1,1) 2s2p²²¹ | 0.5 | -1.00250 | 1.67539 | -1.92408 | -0.02730 | 0.31570 | -1.33054 | -2.29204 | 1.72525 |
|  | (+1,3) 2s2p²²¹ | 0.5 | -1.00963 | 1.74475 | -2.00080 | -0.01195 | 0.24338 | -1.19011 | -2.21953 | 1.63149 |
|  | (+1,1) 2p²¹¹ | 0.5 | -1.00988 | 1.73246 | -2.05540 | -0.02207 | 0.25709 | -1.18404 | -2.19750 | 1.67297 |
|  | (+1,3) 2p²¹¹ | 0.5 | -1.01386 | 1.74997 | -2.03942 | -0.02180 | 0.24779 | -1.15627 | -2.14174 | 1.61475 |
|  | (+1,1) 2p²²⁰ | 0.5 | -0.99670 | 1.67072 | -2.03745 | -0.02000 | 0.27477 | -1.23122 | -2.29458 | 1.75828 |

### **Table S.8.** Optimized parameters for the coefficients of the explicit Boys–Handy correlation function for the elements from Ne to Si using NHF basis set. Values in parenthesis are the charge and multiplicity.

| Atom | System | $c_{1A}$ | $c_{2A}$ | $c_{3A}$ | $c_{4A}$ | $c_{5A}$ | $c_{6A}$ | $c_{7A}$ | $c_{8A}$ | $c_{9A}$ |
| --- | --- | --- | --- | --- | --- | --- | --- | --- | --- | --- |
| Ne | (0,1) 2p²²² | 0.5 | -0.92428 | 1.39601 | -1.73049 | 0.07288 | 0.03456 | -0.68188 | -2.39319 | 1.57139 |
|  | (+1,2) 1s2p²²² | 0.5 | -0.99061 | 1.89850 | -2.16451 | -0.13616 | 0.30308 | -0.99887 | -2.22584 | 1.38515 |
|  | (+1,2) 2s2p²²² | 0.5 | -1.07806 | 1.93372 | -2.14417 | 0.00236 | 0.16278 | -1.07797 | -2.33412 | 1.62646 |
|  | (+1,2) 2p²²¹ | 0.5 | -1.07144 | 1.88881 | -2.18787 | 0.00434 | 0.15974 | -1.02109 | -2.31199 | 1.63231 |
| Na | (0,2) 3s¹ | 0.5 | -1.02079 | 1.71410 | -2.00331 | 0.04683 | 0.01891 | -0.58525 | -2.25024 | 1.54511 |
|  | (+1,1) 1s¹3s¹ | 0.5 | -1.04302 | 2.06984 | -2.29396 | -0.16048 | 0.31116 | -0.92175 | -2.04040 | 1.34032 |
|  | (+1,3) 1s¹3s¹ | 0.5 | -1.06225 | 2.13897 | -2.35338 | -0.16636 | 0.31985 | -0.92979 | -2.02011 | 1.33607 |
|  | (+1,1) 2s¹3s¹ | 0.5 | -1.29032 | 2.66883 | -2.77794 | -0.03210 | 0.12307 | -0.93754 | -2.10265 | 1.57634 |
|  | (+1,3) 2s¹3s¹ | 0.5 | -1.32931 | 2.79708 | -2.87656 | -0.04196 | 0.14697 | -0.96975 | -2.06887 | 1.56881 |
|  | (+1,1) 2p²²¹3s¹ | 0.5 | -1.27411 | 2.57482 | -2.75610 | -0.03270 | 0.13198 | -0.90627 | -2.07064 | 1.57445 |
|  | (+1,3) 2p²²¹3s¹ | 0.5 | -1.29026 | 2.63593 | -2.80446 | -0.03528 | 0.13279 | -0.90275 | -2.04926 | 1.56126 |
|  | (+1,1) 2p²²² | 0.5 | -0.99997 | 1.60846 | -1.93909 | 0.03787 | -0.00093 | -0.63669 | -2.45227 | 1.58775 |
| Mg | (0,1) 3s² | 0.5 | -1.04027 | 1.69242 | -1.99550 | -0.01150 | 0.16531 | -0.74737 | -2.20818 | 1.63572 |
|  | (+1,2) 1s¹3s² | 0.5 | -0.97123 | 1.70815 | -1.98164 | -0.19050 | 0.42934 | -1.05254 | -2.05613 | 1.44465 |
|  | (+1,2) 2s¹3s² | 0.5 | -1.24557 | 2.42242 | -2.53464 | -0.07590 | 0.26179 | -1.07964 | -2.08220 | 1.62836 |
|  | (+1,2) 2p⁵3s² | 0.5 | -1.22221 | 2.28587 | -2.47337 | -0.07302 | 0.26297 | -1.04806 | -2.06735 | 1.63852 |
|  | (+1,2) 2s3s | 0.5 | -1.19842 | 2.29510 | -2.52455 | -0.01252 | 0.05527 | -0.62520 | -2.19438 | 1.54264 |
| Al | (0,2) 3p¹⁰⁰ | 0.5 | -1.04399 | 1.65266 | -1.94703 | -0.05331 | 0.25259 | -0.80943 | -2.11165 | 1.63764 |
|  | (+1,1) 1s¹3p¹⁰⁰ | 0.5 | -0.96714 | 1.62606 | -1.90456 | -0.21103 | 0.46990 | -1.05074 | -1.98481 | 1.46382 |
|  | (+1,3) 1s¹3p¹⁰⁰ | 0.5 | -0.97517 | 1.65660 | -1.92682 | -0.21215 | 0.47075 | -1.04969 | -1.97580 | 1.45590 |
|  | (+1,1) 2s¹3p¹⁰⁰ | 0.5 | -1.22336 | 2.31571 | -2.42357 | -0.09844 | 0.28651 | -1.04193 | -1.99197 | 1.59853 |
|  | (+1,3) 2s¹3p¹⁰⁰ | 0.5 | -1.22545 | 2.32851 | -2.42910 | -0.09869 | 0.28262 | -1.03028 | -1.97376 | 1.58146 |
|  | (+1,1) 2p¹²²3p¹⁰⁰ | 0.5 | -1.20785 | 2.18063 | -2.36485 | -0.08665 | 0.29287 | -1.02897 | -2.04259 | 1.63433 |
|  | (+1,3) 2p¹²²3p¹⁰⁰ | 0.5 | -1.21366 | 2.20577 | -2.37851 | -0.10129 | 0.30504 | -1.04163 | -1.97314 | 1.62010 |
|  | (+1,1) 2p²¹²3p¹⁰⁰ | 0.5 | -1.20921 | 2.18681 | -2.36072 | -0.09798 | 0.29940 | -1.03477 | -1.98535 | 1.62260 |
|  | (+1,3) 2p²¹²3p¹⁰⁰ | 0.5 | -1.20334 | 2.16890 | -2.34239 | -0.10013 | 0.30131 | -1.03362 | -1.97034 | 1.61467 |
|  | (+1,1) 3s⁰3p¹⁰⁰ | 0.5 | -1.18400 | 2.14145 | -2.38707 | -0.06406 | 0.23939 | -0.85114 | -2.17490 | 1.64484 |
|  | (+1,3) 3s⁰3p¹⁰⁰ | 0.5 | -1.22400 | 2.36023 | -2.54690 | -0.05399 | 0.14838 | -0.66369 | -2.02206 | 1.48266 |
|  | (+1,1) 3s² | 0.5 | -1.14240 | 1.99918 | -2.24157 | -0.06562 | 0.23965 | -0.83864 | -2.15296 | 1.61289 |
| Si | (0,3) 3p¹¹⁰ | 0.5 | -1.03494 | 1.56459 | -1.83033 | -0.09403 | 0.34548 | -0.87527 | -1.98748 | 1.59803 |
|  | (+1,2) 1s¹3p¹¹⁰ | 0.5 | -0.96345 | 1.54710 | -1.80800 | -0.23130 | 0.51494 | -1.05632 | -1.89475 | 1.44799 |
|  | (+1,4) 1s¹3p¹¹⁰ | 0.5 | -0.95513 | 1.52637 | -1.78268 | -0.23485 | 0.51864 | -1.05251 | -1.86791 | 1.42787 |
|  | (+1,2) 2s¹3p¹¹⁰ | 0.5 | -1.19395 | 2.17998 | -2.26955 | -0.12374 | 0.32852 | -1.02530 | -1.87674 | 1.53972 |
|  | (+1,4) 2s¹3p¹¹⁰ | 0.5 | -1.19123 | 2.17860 | -2.25503 | -0.12556 | 0.32274 | -1.00741 | -1.83788 | 1.50863 |
|  | (+1,2) 2p¹²²3p¹¹⁰ | 0.5 | -1.18428 | 2.04994 | -2.20801 | -0.11204 | 0.34041 | -1.02588 | -1.93888 | 1.58473 |
|  | (+1,4) 2p¹²²3p¹¹⁰ | 0.5 | -1.18638 | 2.05822 | -2.19933 | -0.12584 | 0.34772 | -1.02992 | -1.85837 | 1.56005 |
|  | (+1,2) 2p²²¹3p¹¹⁰ | 0.5 | -1.18747 | 2.05848 | -2.20126 | -0.12319 | 0.34449 | -1.02882 | -1.87386 | 1.56805 |
|  | (+1,4) 2p²²¹3p¹¹⁰ | 0.5 | -1.18692 | 2.05932 | -2.19660 | -0.12451 | 0.34436 | -1.02423 | -1.85868 | 1.55627 |
|  | (+1,2) 3s¹3p¹¹⁰ | 0.5 | -1.14801 | 1.92384 | -2.17317 | -0.11013 | 0.39324 | -1.02704 | -2.12176 | 1.69285 |
|  | (+1,4) 3s¹3p¹¹⁰ | 0.5 | -1.17409 | 2.14552 | -2.28730 | -0.09668 | 0.26050 | -0.72564 | -1.83238 | 1.38289 |
|  | (+1,2) 3p¹⁰⁰ | 0.5 | -1.14594 | 1.95594 | -2.17865 | -0.10430 | 0.34146 | -0.92216 | -2.03962 | 1.60196 |

### **Table S.9.** Optimized parameters for the coefficients of the explicit Boys–Handy correlation function for the elements from P to Ar using NHF basis set. Values in parenthesis are the charge and multiplicity.

| Atom | System | $c_{1A}$ | $c_{2A}$ | $c_{3A}$ | $c_{4A}$ | $c_{5A}$ | $c_{6A}$ | $c_{7A}$ | $c_{8A}$ | $c_{9A}$ |
| --- | --- | --- | --- | --- | --- | --- | --- | --- | --- | --- |
| P | (0,4) 3p¹¹¹ | 0.5 | -1.02450 | 1.46835 | -1.68350 | -0.12551 | 0.41856 | -0.91859 | -1.85315 | 1.52533 |
|  | (+1,3) 1s¹3p¹¹¹ | 0.5 | -0.95629 | 1.45394 | -1.68095 | -0.24848 | 0.56072 | -1.06576 | -1.79719 | 1.40582 |
|  | (+1,5) 1s¹3p¹¹¹ | 0.5 | -0.95288 | 1.44781 | -1.66748 | -0.25302 | 0.57022 | -1.06916 | -1.77255 | 1.38672 |
|  | (+1,3) 2s¹3p¹¹¹ | 0.5 | -1.17336 | 2.06560 | -2.11985 | -0.14672 | 0.37139 | -1.01510 | -1.75212 | 1.46357 |
|  | (+1,5) 2s¹3p¹¹¹ | 0.5 | -1.17237 | 2.07311 | -2.11428 | -0.14917 | 0.36370 | -0.99694 | -1.70813 | 1.43281 |
|  | (+1,3) 2p¹²²3p¹¹¹ | 0.5 | -1.17767 | 1.97088 | -2.08393 | -0.13210 | 0.38075 | -1.02177 | -1.83445 | 1.52068 |
|  | (+1,5) 2p¹²²3p¹¹¹ | 0.5 | -1.17520 | 1.96470 | -2.05710 | -0.14692 | 0.38622 | -1.02036 | -1.73881 | 1.48576 |
|  | (+1,3) 3s¹3p¹¹¹ | 0.5 | -1.13539 | 1.79432 | -2.01859 | -0.14631 | 0.50948 | -1.15482 | -2.04997 | 1.70073 |
|  | (+1,5) 3s¹3p¹¹¹ | 0.5 | -1.13954 | 1.95829 | -2.05189 | -0.13220 | 0.36579 | -0.80582 | -1.68408 | 1.30740 |
|  | (+1,3) 3p¹¹⁰ | 0.5 | -1.13387 | 1.85542 | -2.04151 | -0.13638 | 0.42631 | -0.98091 | -1.91289 | 1.55229 |
| S | (0,3) 3p²¹¹ | 0.5 | -1.05639 | 1.49579 | -1.68718 | -0.14515 | 0.48573 | -0.99611 | -1.83658 | 1.54663 |
|  | (+1,2) 1s¹3p²¹¹ | 0.5 | -0.99244 | 1.49262 | -1.70812 | -0.25986 | 0.60979 | -1.11305 | -1.79421 | 1.44497 |
|  | (+1,4) 1s¹3p²¹¹ | 0.5 | -0.98465 | 1.46825 | -1.68238 | -0.26215 | 0.61603 | -1.11662 | -1.78208 | 1.43431 |
|  | (+1,2) 2s¹3p²¹¹ | 0.5 | -1.17775 | 2.02142 | -2.07598 | -0.16157 | 0.41183 | -1.04125 | -1.73149 | 1.47732 |
|  | (+1,4) 2s¹3p²¹¹ | 0.5 | -1.18018 | 2.03551 | -2.08405 | -0.16236 | 0.40549 | -1.02677 | -1.70563 | 1.45940 |
|  | (+1,2) 2p¹²²3p²¹¹ | 0.5 | -1.19762 | 1.97448 | -2.07389 | -0.14859 | 0.42519 | -1.05587 | -1.80738 | 1.53265 |
|  | (+1,4) 2p¹²²3p²¹¹ | 0.5 | -1.19794 | 1.97593 | -2.06958 | -0.14849 | 0.42355 | -1.05125 | -1.79892 | 1.52340 |
|  | (+1,2) 2p²¹²3p²¹¹ | 0.5 | -1.20323 | 1.99054 | -2.08611 | -0.14631 | 0.42371 | -1.05605 | -1.81946 | 1.53650 |
|  | (+1,4) 2p²¹²3p²¹¹ | 0.5 | -1.19493 | 1.95612 | -2.04568 | -0.15793 | 0.43028 | -1.06242 | -1.75665 | 1.52396 |
|  | (+1,2) 3s¹3p⁴ | 0.5 | -1.15243 | 1.78253 | -1.97067 | -0.16699 | 0.57483 | -1.21484 | -1.98565 | 1.68540 |
|  | (+1,2) 3s¹3p⁴ | 0.5 | -1.15324 | 1.86305 | -1.99987 | -0.16007 | 0.50044 | -1.03956 | -1.80729 | 1.49993 |
|  | (+1,2) 3p¹¹¹ | 0.5 | -1.15393 | 1.85102 | -2.00833 | -0.15913 | 0.49997 | -1.05802 | -1.85620 | 1.55227 |
|  | (+1,4) 3p¹¹¹ | 0.5 | -1.15506 | 1.86833 | -1.99455 | -0.15977 | 0.48921 | -1.02412 | -1.79553 | 1.49225 |
|  | (+1,2) 3p²¹⁰ | 0.5 | -1.14711 | 1.81909 | -1.99574 | -0.15973 | 0.50589 | -1.07294 | -1.88207 | 1.58220 |
| Cl | (0,2) 3p²²¹ | 0.5 | -1.07369 | 1.48572 | -1.65116 | -0.16018 | 0.53767 | -1.05003 | -1.80467 | 1.54212 |
|  | (+1,1) 1s¹3p²²¹ | 0.5 | -1.02241 | 1.52557 | -1.71578 | -0.26804 | 0.64942 | -1.14512 | -1.76902 | 1.44790 |
|  | (+1,3) 1s¹3p²²¹ | 0.5 | -1.01365 | 1.49656 | -1.68976 | -0.26887 | 0.65138 | -1.14530 | -1.76503 | 1.44445 |
|  | (+1,1) 2s¹3p²²¹ | 0.5 | -1.18810 | 2.00588 | -2.05098 | -0.17304 | 0.44595 | -1.05669 | -1.70010 | 1.46889 |
|  | (+1,3) 2s¹3p²²¹ | 0.5 | -1.18995 | 2.01461 | -2.05490 | -0.17416 | 0.44313 | -1.05136 | -1.68517 | 1.46096 |
|  | (+1,1) 2p¹²²3p²²¹ | 0.5 | -1.21718 | 1.98222 | -2.05814 | -0.15870 | 0.46017 | -1.07912 | -1.78710 | 1.52801 |
|  | (+1,3) 2p¹²²3p²²¹ | 0.5 | -1.21734 | 1.98269 | -2.05487 | -0.15865 | 0.45861 | -1.07535 | -1.77971 | 1.52126 |
|  | (+1,1) 2p²²¹3p²²¹ | 0.5 | -1.21767 | 1.98289 | -2.05606 | -0.15746 | 0.45883 | -1.07731 | -1.79120 | 1.52694 |
|  | (+1,3) 2p²²¹3p²²¹ | 0.5 | -1.22039 | 1.98516 | -2.05178 | -0.16840 | 0.46221 | -1.08268 | -1.73250 | 1.52223 |
|  | (+1,1) 3s¹3p²²¹ | 0.5 | -1.16717 | 1.77445 | -1.91822 | -0.18292 | 0.62302 | -1.25348 | -1.91070 | 1.64804 |
|  | (+1,3) 3s¹3p²²¹ | 0.5 | -1.16581 | 1.80016 | -1.92822 | -0.17956 | 0.59245 | -1.18342 | -1.84516 | 1.57879 |
|  | (+1,1) 3p¹²¹ | 0.5 | -1.16825 | 1.83196 | -1.96479 | -0.17372 | 0.55405 | -1.11171 | -1.81589 | 1.54612 |
|  | (+1,3) 3p¹²¹ | 0.5 | -1.16334 | 1.81899 | -1.93988 | -0.17472 | 0.55276 | -1.10106 | -1.78968 | 1.52046 |
|  | (+1,1) 3p²²⁰ | 0.5 | -1.16663 | 1.81593 | -1.96217 | -0.17420 | 0.56333 | -1.13364 | -1.84609 | 1.57618 |
| Ar | (0,1) 3p²²² | 0.5 | -1.09447 | 1.49752 | -1.62523 | -0.16969 | 0.57657 | -1.08657 | -1.76936 | 1.51668 |
|  | (+1,2) 1s¹3p²²² | 0.5 | -1.04176 | 1.53162 | -1.69255 | -0.27358 | 0.68112 | -1.16888 | -1.73492 | 1.43257 |
|  | (+1,2) 2s¹3p²²² | 0.5 | -1.19710 | 1.98735 | -2.01404 | -0.18273 | 0.47710 | -1.07391 | -1.66479 | 1.45077 |
|  | (+1,2) 2p¹²²3p²²² | 0.5 | -1.23064 | 1.97830 | -2.02950 | -0.16709 | 0.48933 | -1.09374 | -1.75653 | 1.50781 |
|  | (+1,2) 3s¹3p²²² | 0.5 | -1.18670 | 1.78865 | -1.88811 | -0.19415 | 0.65759 | -1.28178 | -1.84663 | 1.61126 |
|  | (+1,2) 3p²²¹ | 0.5 | -1.17684 | 1.80075 | -1.90392 | -0.18692 | 0.59964 | -1.15386 | -1.76292 | 1.52393 |

### **Table S.10.** Calculated energies (a.u.) for the atoms and ions from He to F using SZ, DZ and NHF basis sets.

| Atom | System | SZ | DZ | NHF |
| --- | --- | --- | --- | --- |
| He | (0,1) 1s² | -2.90238 | -2.90319 | -2.90326 |
| Li | (0,2) 2s¹ | -7.47387 | -7.47501 | -7.47479 |
|  | (+1,1) 1s¹2s¹ | -5.06876 | -5.07717 | -5.07747 |
|  | (+1,3) 1s¹2s¹ | -5.10587 | -5.10854 | -5.10769 |
|  | (+1,1) 1s² | -7.27808 | -7.27949 | -7.27950 |
| Be | (0,1) 2s² | -14.63873 | -14.64022 | -14.64048 |
|  | (+1,2) 1s¹2s² | -10.07532 | -10.07852 | -10.07907 |
|  | (+1,2) 2s¹ | -14.31775 | -14.31948 | -14.31956 |
| B | (0,2) 2p¹⁰⁰ | -24.59679 | -24.62084 | -24.62057 |
|  | (+1,1) 1s2p¹⁰⁰ | -17.15678 | -17.18651 | -17.18526 |
|  | (+1,3) 1s2p¹⁰⁰ | -17.17755 | -17.21885 | -17.21917 |
|  | (+1,1) 2s¹2p¹⁰⁰ | -24.06346 | -24.08418 | -24.08458 |
|  | (+1,3) 2s¹2p¹⁰⁰ | -24.14836 | -24.16662 | -24.16737 |
|  | (+1,1) 2s² | -24.30535 | -24.30856 | -24.30985 |
| C | (0,3) 2p¹¹⁰ | -37.74432 | -37.80534 | -37.80677 |
|  | (+1,2) 1s¹2p¹¹⁰ | -26.76606 | -26.82503 | -26.82604 |
|  | (+1,4) 1s¹2p¹¹⁰ | -26.82244 | -26.90374 | -26.90563 |
|  | (+1,2) 2s¹2p¹¹⁰ | -36.96406 | -37.01389 | -37.01426 |
|  | (+1,4) 2s¹2p¹¹⁰ | -37.16255 | -37.22049 | -37.22135 |
|  | (+1,2) 2p¹⁰⁰ | -37.35104 | -37.38509 | -37.38625 |
| N | (0,4) 2p¹¹¹ | -54.43473 | -54.54620 | -54.54760 |
|  | (+1,3) 1s¹2p¹¹¹ | -39.23961 | -39.33523 | -39.33971 |
|  | (+1,5) 1s¹2p¹¹¹ | -39.34643 | -39.47804 | -39.48214 |
|  | (+1,3) 2s¹2p¹¹¹ | -53.34617 | -53.45098 | -53.45106 |
|  | (+1,5) 2s¹2p¹¹¹ | -53.72315 | -53.82442 | -53.82531 |
|  | (+1,3) 2p¹¹⁰ | -53.91720 | -54.00505 | -54.00668 |
| O | (0,3) 2p²¹¹ | -74.83374 | -75.01015 | -75.01702 |
|  | (+1,2) 1s¹2p²¹¹ | -54.71160 | -54.89302 | -54.89261 |
|  | (+1,4) 1s¹2p²¹¹ | -54.80245 | -54.99650 | -55.00088 |
|  | (+1,2) 2s¹2p²¹¹ | -73.52052 | -73.68182 | -73.68396 |
|  | (+1,4) 2s¹2p²¹¹ | -73.79887 | -73.96740 | -73.97233 |
|  | (+1,2) 2p¹¹¹ | -74.29513 | -74.43622 | -74.43946 |
|  | (+1,4) 2p¹¹¹ | -74.35385 | -74.51495 | -74.51593 |
|  | (+1,2) 2p²¹⁰ | -74.19936 | -74.35558 | -74.35820 |
| F | (0,2) 2p²²¹ | -99.40971 | -99.66982 | -99.68345 |
|  | (+1,1) 1s2p²²¹ | -73.69063 | -73.96585 | -73.97413 |
|  | (+1,3) 1s2p²²¹ | -73.74909 | -74.02628 | -74.03299 |
|  | (+1,1) 2s2p²²¹ | -97.86024 | -98.09535 | -98.10078 |
|  | (+1,3) 2s2p²²¹ | -98.02247 | -98.25827 | -98.26479 |
|  | (+1,1) 2p²¹¹ | -98.75206 | -98.98761 | -98.99248 |
|  | (+1,3) 2p²¹¹ | -98.80104 | -99.03125 | -99.03719 |
|  | (+1,1) 2p²²⁰ | -98.64944 | -98.89670 | -98.90100 |

### **Table S.11.** Calculated energies (a.u.) for the atoms and ions from Ne to Si using SZ, DZ and NHF basis sets.

| Atom | System | SZ | DZ | NHF |
| --- | --- | --- | --- | --- |
| Ne | (0,1) 2p²²² | -128.51146 | -128.86825 | -128.88419 |
|  | (+1,2) 1s2p²²² | -96.53798 | -96.91120 | -96.92429 |
|  | (+1,2) 2s2p²²² | -126.72904 | -127.04197 | -127.05250 |
|  | (+1,2) 2p²²¹ | -127.74495 | -128.07910 | -128.08738 |
| Na | (0,2) 3s¹ | -161.74251 | -162.18275 | -162.19896 |
|  | (+1,1) 1s¹3s¹ | -122.04312 | -122.56160 | -122.56792 |
|  | (+1,3) 1s¹3s¹ | -122.06598 | -122.56591 | -122.57285 |
|  | (+1,1) 2s¹3s¹ | -159.04899 | -159.52922 | -159.53525 |
|  | (+1,3) 2s¹3s¹ | -159.07743 | -159.54459 | -159.55014 |
|  | (+1,1) 2p²²¹3s¹ | -160.30512 | -160.77338 | -160.78085 |
|  | (+1,3) 2p²²¹3s¹ | -160.30868 | -160.77965 | -160.78729 |
|  | (+1,1) 2p²²² | -161.55968 | -162.00414 | -162.01246 |
| Mg | (0,1) 3s² | -199.38326 | -199.96747 | -199.97257 |
|  | (+1,2) 1s¹3s² | -151.23490 | -151.82708 | -151.83513 |
|  | (+1,2) 2s¹3s² | -195.82956 | -196.38738 | -196.38998 |
|  | (+1,2) 2p⁵3s² | -197.28937 | -197.83498 | -197.83921 |
|  | (+1,2) 2s3s | -199.15496 | -199.69844 | -199.71087 |
| Al | (0,2) 3p¹⁰⁰ | -241.62177 | -242.25256 | -242.25122 |
|  | (+1,1) 1s¹3p¹⁰⁰ | -184.10241 | -184.68438 | -184.68523 |
|  | (+1,3) 1s¹3p¹⁰⁰ | -184.12972 | -184.68910 | -184.68544 |
|  | (+1,1) 2s¹3p¹⁰⁰ | -237.03124 | -237.57092 | -237.56640 |
|  | (+1,3) 2s¹3p¹⁰⁰ | -237.05534 | -237.57322 | -237.57375 |
|  | (+1,1) 2p¹²²3p¹⁰⁰ | -238.65889 | -239.21645 | -239.21694 |
|  | (+1,3) 2p¹²²3p¹⁰⁰ | -238.69597 | -239.24064 | -239.24371 |
|  | (+1,1) 2p²¹²3p¹⁰⁰ | -238.68381 | -239.22214 | -239.22640 |
|  | (+1,3) 2p²¹²3p¹⁰⁰ | -238.68136 | -239.22648 | -239.22775 |
|  | (+1,1) 3s⁰3p¹⁰⁰ | -241.23276 | -241.82702 | -241.82493 |
|  | (+1,3) 3s⁰3p¹⁰⁰ | -241.30276 | -241.88171 | -241.88352 |
|  | (+1,1) 3s² | -241.45234 | -242.03672 | -242.03774 |
| Si | (0,3) 3p¹¹⁰ | -288.58074 | -289.25163 | -289.25416 |
|  | (+1,2) 1s¹3p¹¹⁰ | -220.80435 | -221.41705 | -221.41132 |
|  | (+1,4) 1s¹3p¹¹⁰ | -220.80342 | -221.42929 | -221.43125 |
|  | (+1,2) 2s¹3p¹¹⁰ | -282.77445 | -283.35295 | -283.35878 |
|  | (+1,4) 2s¹3p¹¹⁰ | -282.81776 | -283.37002 | -283.36513 |
|  | (+1,2) 2p¹²²3p¹¹⁰ | -284.60054 | -285.21445 | -285.21232 |
|  | (+1,4) 2p¹²²3p¹¹⁰ | -284.66402 | -285.24977 | -285.25221 |
|  | (+1,2) 2p²²¹3p¹¹⁰ | -284.63780 | -285.22118 | -285.22079 |
|  | (+1,4) 2p²²¹3p¹¹⁰ | -284.64765 | -285.23498 | -285.23165 |
|  | (+1,2) 3s¹3p¹¹⁰ | -288.03889 | -288.64778 | -288.64653 |
|  | (+1,4) 3s¹3p¹¹⁰ | -288.17938 | -288.78372 | -288.78049 |
|  | (+1,2) 3p¹⁰⁰ | -288.27974 | -288.95513 | -288.95077 |

### **Table S.12.** Calculated energies (a.u.) for the atoms and ions from P to Ar using SZ, DZ and NHF basis sets.

| Atom | System | SZ | DZ | NHF |
| --- | --- | --- | --- | --- |
| P | (0,4) 3p¹¹¹ | -339.41486 | -341.13731 | -341.13748 |
|  | (+1,3) 1s¹3p¹¹¹ | -260.17345 | -262.18281 | -262.18153 |
|  | (+1,5) 1s¹3p¹¹¹ | -260.20206 | -262.21011 | -262.21274 |
|  | (+1,3) 2s¹3p¹¹¹ | -332.37242 | -333.91266 | -333.92079 |
|  | (+1,5) 2s¹3p¹¹¹ | -332.44946 | -333.94146 | -333.94459 |
|  | (+1,3) 2p¹²²3p¹¹¹ | -334.31600 | -335.98610 | -335.98908 |
|  | (+1,5) 2p¹²²3p¹¹¹ | -334.35165 | -336.03416 | -336.03115 |
|  | (+1,3) 3s¹3p¹¹¹ | -338.63618 | -340.33438 | -340.33122 |
|  | (+1,5) 3s¹3p¹¹¹ | -338.89178 | -340.57669 | -340.56799 |
|  | (+1,3) 3p¹¹⁰ | -339.02472 | -340.75226 | -340.74528 |
| S | (0,3) 3p²¹¹ | -397.22698 | -397.96396 | -397.96983 |
|  | (+1,2) 1s¹3p²¹¹ | -306.33780 | -307.03738 | -307.03828 |
|  | (+1,4) 1s¹3p²¹¹ | -306.37020 | -307.06259 | -307.06298 |
|  | (+1,2) 2s¹3p²¹¹ | -388.75443 | -389.29892 | -389.30043 |
|  | (+1,4) 2s¹3p²¹¹ | -388.74410 | -389.31821 | -389.32198 |
|  | (+1,2) 2p¹²²3p²¹¹ | -390.96850 | -391.60447 | -391.59763 |
|  | (+1,4) 2p¹²²3p²¹¹ | -390.99945 | -391.61924 | -391.60817 |
|  | (+1,2) 2p²¹²3p²¹¹ | -390.93987 | -391.57860 | -391.57889 |
|  | (+1,4) 2p²¹²3p²¹¹ | -390.97716 | -391.62930 | -391.63074 |
|  | (+1,2) 3s¹3p⁴ | -396.31510 | -397.03303 | -397.02503 |
|  | (+1,2) 3s¹3p⁴ | -396.48837 | -397.20411 | -397.20603 |
|  | (+1,2) 3p¹¹¹ | -396.82382 | -397.54608 | -397.55816 |
|  | (+1,4) 3p¹¹¹ | -396.87383 | -397.59375 | -397.60236 |
|  | (+1,2) 3p²¹⁰ | -396.81027 | -397.49786 | -397.50083 |
| Cl | (0,2) 3p²²¹ | -459.20753 | -460.00251 | -460.00433 |
|  | (+1,1) 1s¹3p²²¹ | -355.50065 | -356.22819 | -356.23183 |
|  | (+1,3) 1s¹3p²²¹ | -355.52210 | -356.24235 | -356.24087 |
|  | (+1,1) 2s¹3p²²¹ | -449.13585 | -449.75708 | -449.75625 |
|  | (+1,3) 2s¹3p²²¹ | -449.19655 | -449.77231 | -449.77448 |
|  | (+1,1) 2p¹²²3p²²¹ | -451.57841 | -452.27548 | -452.27513 |
|  | (+1,3) 2p¹²²3p²²¹ | -451.60388 | -452.28027 | -452.27704 |
|  | (+1,1) 2p²²¹3p²²¹ | -451.57303 | -452.24330 | -452.24357 |
|  | (+1,3) 2p²²¹3p²²¹ | -451.64575 | -452.29275 | -452.29713 |
|  | (+1,1) 3s¹3p²²¹ | -458.14430 | -458.91090 | -458.91829 |
|  | (+1,3) 3s¹3p²²¹ | -458.24307 | -459.00577 | -459.01434 |
|  | (+1,1) 3p¹²¹ | -458.74683 | -459.49725 | -459.49677 |
|  | (+1,3) 3p¹²¹ | -458.74070 | -459.52341 | -459.52933 |
|  | (+1,1) 3p²²⁰ | -458.68057 | -459.44251 | -459.44437 |
| Ar | (0,1) 3p²²² | -526.51654 | -527.39214 | -527.38377 |
|  | (+1,2) 1s¹3p²²² | -409.19794 | -409.94942 | -409.95256 |
|  | (+1,2) 2s¹3p²²² | -514.86721 | -515.46871 | -515.47129 |
|  | (+1,2) 2p¹²²3p²²² | -517.48169 | -518.18655 | -518.19701 |
|  | (+1,2) 3s¹3p²²² | -525.34895 | -526.15658 | -526.16985 |
|  | (+1,2) 3p²²¹ | -525.99611 | -526.79128 | -526.80431 |

### **Table S.13.** Optimized parameters for the coefficients of the explicit Boys–Handy correlation function for H_2_O and CO using SZ basis set. Values in parenthesis are the charge and multiplicity.

| Molecule | System | $c_{1A}$ | $c_{2A}$ | $c_{3A}$ | $c_{4A}$ | Atom | $c_{5A}$ | $c_{6A}$ | $c_{7A}$ | $c_{8A}$ | $c_{9A}$ |
| --- | --- | --- | --- | --- | --- | --- | --- | --- | --- | --- | --- |
| H_2_O | (0,1) | 0.5 | -1.49550 | 3.30562 | -3.27315 | O | 0.03675 | -0.24326 | -0.31739 | -1.95351 | 1.70380 |
|  |  |  |  |  |  | H | -0.12287 | -0.12207 | 0.17026 | 0.23803 | 0.00118 |
|  | (+1,2) 1a_1_ | 0.5 | -0.60834 | 0.98659 | -0.06897 | O | -0.41607 | 0.53758 | -1.65553 | -1.46849 | 1.60556 |
|  |  |  |  |  |  | H | -0.84103 | -0.16885 | -0.04932 | 2.19419 | -0.16249 |
|  | (+1,2) 2a_1_ | 0.5 | -1.11523 | 2.60576 | -2.73928 | O | -0.03395 | -0.06803 | -0.54496 | -1.92956 | 1.61293 |
|  |  |  |  |  |  | H | 0.83696 | -0.38713 | 0.23468 | -1.46283 | -0.11108 |
|  | (+1,2) 1b_2_ | 0.5 | -0.64420 | 1.63392 | -1.88527 | O | -0.02807 | -0.08676 | -0.59663 | -1.80783 | 1.61576 |
|  |  |  |  |  |  | H | -0.03517 | -0.19299 | 0.17444 | 0.20942 | -0.10524 |
|  | (+1,2) 3a_1_ | 0.5 | -0.93794 | 2.61996 | -2.91648 | O | 0.00428 | -0.17289 | -0.48521 | -1.97714 | 1.73160 |
|  |  |  |  |  |  | H | 0.28250 | -0.35778 | 0.33329 | -0.34382 | -0.14160 |
|  | (+1,2) 1b_1_ | 0.5 | -0.78556 | 2.27701 | -2.60369 | O | 0.09051 | -0.24588 | -0.29580 | -2.08565 | 1.57585 |
|  |  |  |  |  |  | H | 0.69119 | -0.13334 | 0.06341 | -1.29185 | -0.17880 |
| CO | (0,1) | 0.5 | -0.62631 | 0.54424 | -0.16351 | C | 0.08383 | -0.39403 | 0.22719 | -0.28683 | 0.14796 |
|  |  |  |  |  |  | O | 0.10992 | -0.25795 | 0.09767 | -0.70977 | 0.23250 |
|  | (+1,2) 1σ | 0.5 | -0.51305 | 0.87152 | -0.38722 | C | -0.00317 | -0.33332 | 0.18922 | 0.01522 | 0.00411 |
|  |  |  |  |  |  | O | 0.08288 | -0.38334 | 0.23431 | -0.69921 | 0.06873 |
|  | (+1,2) 2σ | 0.5 | -0.49589 | 0.95384 | -0.46782 | C | 0.50221 | -0.92499 | 0.67438 | -0.90583 | -0.11224 |
|  |  |  |  |  |  | O | 0.20829 | -0.35210 | 0.21284 | -0.84806 | 0.10286 |
|  | (+1,2) 3σ | 0.5 | -0.61073 | 0.63071 | 0.08514 | C | -0.25049 | -0.27629 | 0.03261 | 0.53580 | 0.07650 |
|  |  |  |  |  |  | O | -0.27454 | -0.02056 | -0.23734 | 0.19868 | 0.17541 |
|  | (+1,2) 4σ | 0.5 | -0.49705 | 0.38393 | 0.06569 | C | 0.16969 | -0.39166 | 0.20274 | -0.40585 | 0.03515 |
|  |  |  |  |  |  | O | 0.11613 | -0.16621 | -0.04566 | -0.73080 | 0.15121 |
|  | (+1,2) 1π | 0.5 | -0.43882 | 0.33589 | -0.01644 | C | 0.28567 | -0.36044 | 0.18849 | -0.68937 | 0.02058 |
|  |  |  |  |  |  | O | 0.14605 | -0.21175 | 0.03723 | -0.76645 | 0.14110 |
|  | (+1,2) 5σ | 0.5 | -0.48034 | 0.25643 | 0.16332 | C | 0.03124 | -0.27934 | 0.08772 | -0.17877 | 0.10154 |
|  |  |  |  |  |  | O | 0.11390 | -0.26787 | 0.09329 | -0.64847 | 0.11431 |

### **Table S.14.** Optimized parameters for the coefficients of the explicit Boys–Handy correlation function for H_2_O and CO using DZ basis set. Values in parenthesis are the charge and multiplicity.

| Molecule | System | $c_{1A}$ | $c_{2A}$ | $c_{3A}$ | $c_{4A}$ | Atom | $c_{5A}$ | $c_{6A}$ | $c_{7A}$ | $c_{8A}$ | $c_{9A}$ |
| --- | --- | --- | --- | --- | --- | --- | --- | --- | --- | --- | --- |
| H_2_O | (0,1) | 0.5 | -0.56206 | 0.98506 | -1.26808 | O | 0.02673 | 0.38154 | -1.05550 | -2.10076 | 1.57309 |
|  |  |  |  |  |  | H | -0.12765 | -0.03288 | 0.07730 | 0.23565 | -0.12937 |
|  | (+1,2) 1a_1_ | 0.5 | -0.71913 | 1.67081 | -1.88752 | O | -0.08298 | 0.52635 | -1.20375 | -2.18461 | 1.42800 |
|  |  |  |  |  |  | H | 0.05612 | 0.01984 | 0.02405 | -0.16466 | -0.12851 |
|  | (+1,2) 2a_1_ | 0.5 | -0.50705 | 1.16459 | -1.58450 | O | 0.04760 | 0.41602 | -1.15087 | -2.27521 | 1.59744 |
|  |  |  |  |  |  | H | 0.47255 | -0.10527 | 0.07342 | -0.91943 | -0.18126 |
|  | (+1,2) 1b_2_ | 0.5 | -0.63972 | 1.25169 | -1.58819 | O | -0.00816 | 0.45512 | -1.20531 | -2.08488 | 1.58032 |
|  |  |  |  |  |  | H | 0.04618 | -0.01284 | 0.05222 | -0.16228 | -0.12014 |
|  | (+1,2) 3a_1_ | 0.5 | -0.55466 | 1.18813 | -1.51281 | O | -0.01906 | 0.51465 | -1.32165 | -2.11884 | 1.61976 |
|  |  |  |  |  |  | H | 0.00555 | -0.05344 | 0.09214 | -0.00636 | -0.16921 |
|  | (+1,2) 1b_1_ | 0.5 | -0.67702 | 1.55459 | -1.94616 | O | 0.00126 | 0.49227 | -1.27757 | -2.23698 | 1.70610 |
|  |  |  |  |  |  | H | 0.31884 | -0.08152 | 0.07403 | -0.62870 | -0.16208 |
| CO | (0,1) | 0.5 | -0.56229 | 0.75582 | -0.29846 | C | 0.00643 | -0.10734 | -0.03246 | -0.10757 | 0.01939 |
|  |  |  |  |  |  | O | 0.11766 | -0.02403 | -0.17478 | -0.73561 | 0.18354 |
|  | (+1,2) 1σ | 0.5 | -0.17832 | 0.28445 | 0.10106 | C | 0.01933 | -0.03847 | -0.08868 | -0.04682 | -0.13774 |
|  |  |  |  |  |  | O | 0.11081 | -0.01916 | -0.17924 | -0.80585 | 0.00023 |
|  | (+1,2) 2σ | 0.5 | -0.56694 | 1.39096 | -0.58678 | C | -0.07175 | -0.31476 | 0.11090 | 0.25108 | -0.20805 |
|  |  |  |  |  |  | O | 0.15031 | -0.00717 | -0.21280 | -0.81946 | 0.13076 |
|  | (+1,2) 3σ | 0.5 | -0.34456 | 0.33354 | 0.12234 | C | 0.12612 | -0.01921 | -0.15617 | -0.34748 | -0.09230 |
|  |  |  |  |  |  | O | 0.15953 | 0.08843 | -0.33444 | -0.85418 | 0.09163 |
|  | (+1,2) 4σ | 0.5 | -0.41530 | 0.55104 | -0.04308 | C | 0.05829 | -0.08203 | -0.07649 | -0.14920 | -0.09760 |
|  |  |  |  |  |  | O | 0.14056 | 0.04936 | -0.30365 | -0.79196 | 0.10555 |
|  | (+1,2) 1π | 0.5 | -0.43882 | 0.33589 | -0.01644 | C | 0.28567 | -0.36044 | 0.18849 | -0.68937 | 0.02058 |
|  |  |  |  |  |  | O | 0.14605 | -0.21175 | 0.03723 | -0.76645 | 0.14110 |
|  | (+1,2) 5σ | 0.5 | -0.48593 | 0.69503 | -0.14917 | C | 0.01813 | -0.05931 | -0.12700 | -0.11108 | -0.02649 |
|  |  |  |  |  |  | O | 0.11782 | -0.01561 | -0.18853 | -0.67635 | 0.05517 |

### **Table S.15.** Optimized parameters for the coefficients of the explicit Boys–Handy correlation function for H_2_O and CO using NHF basis set. Values in parenthesis are the charge and multiplicity.

| Molecule | System | $c_{1A}$ | $c_{2A}$ | $c_{3A}$ | $c_{4A}$ | Atom | $c_{5A}$ | $c_{6A}$ | $c_{7A}$ | $c_{8A}$ | $c_{9A}$ |
| --- | --- | --- | --- | --- | --- | --- | --- | --- | --- | --- | --- |
| H_2_O | (0,1) | 0.5 | -0.47364 | 0.52408 | -1.10735 | O | 0.13409 | 0.14418 | -0.66692 | -2.23810 | 1.48949 |
|  |  |  |  |  |  | H | 0.53922 | -0.25181 | 0.14160 | -0.92650 | -0.06421 |
|  | (+1,2) 1a_1_ | 0.5 | -0.82837 | 1.77420 | -1.93291 | O | -0.03826 | 0.29072 | -0.94970 | -2.10034 | 1.36032 |
|  |  |  |  |  |  | H | 0.06403 | -0.06541 | 0.01758 | -0.01562 | -0.07190 |
|  | (+1,2) 2a_1_ | 0.5 | -0.64741 | 1.11965 | -1.34735 | O | 0.00857 | 0.41469 | -1.23383 | -2.11447 | 1.57295 |
|  |  |  |  |  |  | H | 0.01853 | -0.06536 | 0.00513 | 0.08444 | -0.10306 |
|  | (+1,2) 1b_2_ | 0.5 | -0.69750 | 1.29233 | -1.53118 | O | 0.01434 | 0.36066 | -1.12154 | -2.06989 | 1.54252 |
|  |  |  |  |  |  | H | -0.00665 | -0.07460 | 0.02682 | 0.12609 | -0.10011 |
|  | (+1,2) 3a_1_ | 0.5 | -0.66524 | 1.17389 | -1.44861 | O | 0.01169 | 0.37446 | -1.16217 | -2.07227 | 1.54983 |
|  |  |  |  |  |  | H | -0.01250 | -0.10367 | 0.06171 | 0.16604 | -0.09745 |
|  | (+1,2) 1b_1_ | 0.5 | -0.68245 | 1.15979 | -1.43070 | O | 0.01232 | 0.38313 | -1.17417 | -2.06960 | 1.54492 |
|  |  |  |  |  |  | H | -0.05026 | -0.04912 | 0.01383 | 0.20904 | -0.08704 |
| CO | (0,1) | 0.5 | -0.67406 | 1.04973 | -0.52863 | C | -0.02422 | -0.17070 | 0.03381 | -0.01781 | 0.03282 |
|  |  |  |  |  |  | O | 0.11861 | -0.10434 | -0.09824 | -0.70607 | 0.20036 |
|  | (+1,2) 1σ | 0.5 | -0.29607 | 0.61598 | -0.18404 | C | 0.03340 | -0.10221 | -0.01895 | -0.06617 | -0.12315 |
|  |  |  |  |  |  | O | 0.14211 | -0.15600 | -0.04470 | -0.81134 | 0.01316 |
|  | (+1,2) 2σ | 0.5 | -0.51530 | 1.31890 | -0.65283 | C | 0.16514 | -0.44322 | 0.25218 | -0.25545 | -0.21435 |
|  |  |  |  |  |  | O | 0.14384 | -0.11905 | -0.07426 | -0.67450 | 0.05432 |
|  | (+1,2) 3σ | 0.5 | -0.45535 | 0.65231 | -0.14790 | C | 0.14790 | -0.08567 | -0.08193 | -0.38155 | -0.08188 |
|  |  |  |  |  |  | O | 0.18300 | -0.01442 | -0.22630 | -0.86163 | 0.09528 |
|  | (+1,2) 4σ | 0.5 | -0.59319 | 1.00274 | -0.38412 | C | -0.00870 | -0.15134 | -0.00594 | 0.02700 | -0.07043 |
|  |  |  |  |  |  | O | 0.12651 | -0.06052 | -0.20680 | -0.70088 | 0.13082 |
|  | (+1,2) 1π | 0.5 | -0.44321 | 0.70836 | -0.33006 | C | 0.25387 | -0.16692 | 0.02980 | -0.60434 | -0.08652 |
|  |  |  |  |  |  | O | 0.23020 | -0.07966 | -0.12078 | -0.97201 | 0.10603 |
|  | (+1,2) 5σ | 0.5 | -0.49636 | 0.73369 | -0.21964 | C | 0.06682 | -0.12326 | -0.05380 | -0.21034 | -0.02003 |
|  |  |  |  |  |  | O | 0.16434 | -0.10591 | -0.08622 | -0.75769 | 0.05237 |

### **Table S.16.** Optimized parameters for the coefficients of the explicit Boys–Handy correlation function for H_2_O and CO using NHF_SP basis set. Values in parenthesis are the charge and multiplicity.

| Molecule | System | $c_{1A}$ | $c_{2A}$ | $c_{3A}$ | $c_{4A}$ | Atom | $c_{5A}$ | $c_{6A}$ | $c_{7A}$ | $c_{8A}$ | $c_{9A}$ |
| --- | --- | --- | --- | --- | --- | --- | --- | --- | --- | --- | --- |
| H_2_O | (0,1) | 0.5 | -0.81165 | 1.23048 | -1.48765 | O | 0.09318 | 0.18629 | -0.76936 | -2.07907 | 1.44439 |
|  |  |  |  |  |  | H | 0.06076 | -0.13470 | 0.08589 | -0.10192 | -0.01080 |
|  | (+1,2) 1a_1_ | 0.5 | -1.03240 | 2.10563 | -2.18616 | O | -0.03740 | 0.25873 | -0.89091 | -2.04975 | 1.33625 |
|  |  |  |  |  |  | H | 0.04357 | -0.02071 | -0.03330 | -0.09600 | -0.01119 |
|  | (+1,2) 2a_1_ | 0.5 | -0.76894 | 1.36879 | -1.54954 | O | 0.01238 | 0.39607 | -1.17206 | -2.06945 | 1.53602 |
|  |  |  |  |  |  | H | 0.07301 | -0.07013 | -0.01840 | -0.07414 | -0.07673 |
|  | (+1,2) 1b_2_ | 0.5 | -0.70154 | 1.41920 | -1.67625 | O | 0.02010 | 0.30594 | -1.01293 | -2.03696 | 1.53304 |
|  |  |  |  |  |  | H | 0.11275 | -0.14327 | 0.06550 | -0.11721 | -0.11567 |
|  | (+1,2) 3a_1_ | 0.5 | -0.82579 | 1.56186 | -1.75992 | O | 0.02268 | 0.30612 | -1.04562 | -1.99194 | 1.47413 |
|  |  |  |  |  |  | H | 0.04745 | -0.15746 | 0.09809 | -0.00007 | -0.06355 |
|  | (+1,2) 1b_1_ | 0.5 | -0.86578 | 1.56433 | -1.75621 | O | 0.01797 | 0.34405 | -1.10027 | -2.01065 | 1.49634 |
|  |  |  |  |  |  | H | 0.01282 | -0.06291 | 0.01421 | 0.00482 | -0.04783 |
| CO | (0,1) | 0.5 | -0.66271 | 0.83494 | -0.41222 | C | 0.06712 | -0.07085 | -0.08503 | -0.34039 | 0.08779 |
|  |  |  |  |  |  | O | 0.17024 | -0.04786 | -0.16079 | -0.92210 | 0.25056 |
|  | (+1,2) 1σ | 0.5 | -0.37865 | 0.57371 | -0.18737 | C | 0.02208 | 0.03487 | -0.18384 | -0.17741 | -0.04811 |
|  |  |  |  |  |  | O | 0.15147 | -0.09814 | -0.12494 | -0.94887 | 0.11586 |
|  | (+1,2) 2σ | 0.5 | -0.65756 | 1.39313 | -0.73539 | C | 0.15361 | -0.32186 | 0.11457 | -0.38399 | -0.10474 |
|  |  |  |  |  |  | O | 0.15031 | -0.00717 | -0.21280 | -0.81946 | 0.13076 |
|  | (+1,2) 3σ | 0.5 | -0.57963 | 0.74584 | -0.21239 | C | 0.05031 | 0.03717 | -0.22554 | -0.26461 | -0.01244 |
|  |  |  |  |  |  | O | 0.16060 | 0.05636 | -0.32803 | -0.90012 | 0.17905 |
|  | (+1,2) 4σ | 0.5 | -0.55319 | 0.73265 | -0.23059 | C | 0.06456 | -0.04381 | -0.13135 | -0.25997 | -0.01576 |
|  |  |  |  |  |  | O | 0.16804 | 0.00648 | -0.27463 | -0.89581 | 0.17194 |
|  | (+1,2) 1π | 0.5 | -0.57555 | 0.81704 | -0.34669 | C | 0.04209 | -0.02135 | -0.13632 | -0.21715 | -0.01570 |
|  |  |  |  |  |  | O | 0.14782 | -0.00280 | -0.24389 | -0.83864 | 0.18229 |
|  | (+1,2) 5σ | 0.5 | -0.60415 | 0.83641 | -0.30714 | C | 0.05320 | -0.03587 | -0.16020 | -0.26871 | 0.03107 |
|  |  |  |  |  |  | O | 0.16724 | -0.01560 | -0.20554 | -0.87547 | 0.12889 |

### **Table S.17.** Calculated energies for H_2_O and CO using SZ, DZ, NHF and NHF_SP basis sets.

| Molecule | System | SZ | DZ | NHF | NHF_SP |
| --- | --- | --- | --- | --- | --- |
| H_2_O | (0,1) | -76.13867 | -76.35040 | -76.36479 | -76.34403 |
|  | (+1,2) 1a_1_ | -56.25318 | -56.51128 | -56.53440 | -56.51787 |
|  | (+1,2) 2a_1_ | -74.86596 | -75.08421 | -75.10425 | -75.08172 |
|  | (+1,2) 1b_2_ | -75.47484 | -75.66606 | -75.67498 | -75.63695 |
|  | (+1,2) 3a_1_ | -75.57777 | -75.80340 | -75.82167 | -75.81915 |
|  | (+1,2) 1b_1_ | -75.65394 | -75.88770 | -75.90143 | -75.88543 |
| CO | (0,1) | -112.87946 | -113.15648 | -113.16507 | -113.16452 |
|  | (+1,2) 1σ | -92.88114 | -93.20038 | -93.21741 | -93.18967 |
|  | (+1,2) 2σ | -101.97007 | -102.19715 | -102.21988 | -102.20048 |
|  | (+1,2) 3σ | -111.52833 | -111.70760 | -111.71438 | -111.68847 |
|  | (+1,2) 4σ | -112.13276 | -112.40551 | -112.41442 | -112.39363 |
|  | (+1,2) 1π | -112.25972 | -112.51872 | -112.52183 | -112.53183 |
|  | (+1,2) 5σ | -112.36557 | -112.61921 | -112.63577 | -112.60947 |
